# Supplementary material for: OTU Deubiquitinases Reveal Mechanisms of Linkage Specificity and Enable Ubiquitin Chain Restriction Analysis
Source: Cell. 2013 Jul 3;154(1):169–84. doi: 10.1016/j.cell.2013.05.046 (PMC3705208; doi:10.1016/j.cell.2013.05.046)
Supplement: Table S1. Crystallization Data Collection and Refinement Statistics, Related to Figures 5 and 6 — Values in parentheses are for the highest resolution shell. [file mmc1.pdf]

Table S1. **Crystallization Data Collection and Refinement Statistics, Related to Figure 5 and 6**

	OTUD1 OTU (287-437)	OTUD2 OTU (132-314)	OTUD3 cat (52-209)	OTUD2 OTU C160A (132-314) Lys11 diUb	OTUD2 OTU C160A (147-314) K11 peptide
**Data collection statistics**					
Beamline	ESRF ID23-1	ESRF ID23-1	ESRF ID29	Diamond I-03	Diamond I-04
Wavelength (Å)	0.9794	0.9792	0.97627	0.9763	0.9464
Space Group	$I4$	$P3_121$	$P3_212$	$C2$	$P6$
Unit Cell (Å)	a = 82.24<br>b = 82.24<br>c = 103.94	a = 60.67<br>b = 60.67<br>c = 86.18	a = 36.20<br>b = 36.20<br>c = 211.05	a = 174.79<br>b = 44.22<br>c = 84.95<br>β = 91.4º	a = 164.48<br>b = 164.48<br>c = 44.73
Resolution (Å)	52.0 – 2.10 (2.21 – 2.10)	33.3-1.47 (1.55-1.47)	42.21-1.55 (1.63-1.55)	50.0 - 3.03 (3.19 - 3.03)	53.84 - 2.35 (2.43 - 2.35)
Observed reflections	65317 (9688)	128709 (18568)	92022 (13007)	40480 (6177)	177275 (17419)
Unique reflections	20153 (2957)	30915 (4562)	23429 (3352)	12691 (1860)	29294 (2827)
Redundancy	3.2 (3.3)	4.2 (4.1)	3.9 (3.9)	3.2 (3.3)	6.1 (6.2)
Completeness (%)	99.8 (99.9)	97.2 (99.5)	98.9 (99.1)	97.9 (98.8)	100 (100)
R_{merge}	0.095 (0.530)	0.069 (0.623)	0.041 (0.477)	0.135 (0.441)	0.118 (0.887)
$\langle I/\sigma I \rangle$	8.9 (2.3)	10.5 (2.0)	14.9 (2.1)	7.1 (3.3)	10.0 (1.9)
**Phasing statistics**					
Molecular Replacement	Phaser	Phaser	Phaser	Phaser	Phaser
Search model	OTUD3 OTU	3by4 (yOtu1)	OTUD5 OTU (without N-terminal helix)	OTUD2 OTU, Ub	OTUD2 OTU, Ub
**Refinement statistics**					
Reflections in test set	1029	1554	1204	613	1487
R_{cryst}	16.2	17.9	17.5	19.4	18.7
R_{free}	20.2	19.5	22.9	25.4	23.7
**Number of groups**					
Protein residues	299	175	141	546	485
Ions and ligand atoms	10	24	0	2	5
Water	147	131	113	11	231
Wilson B-factor	25.7	18.3	23.66	42.89	37.2
**RMSD from ideal geometry**					
Bond length (Å)	0.007	0.020	0.005	0.009	0.009
Bond angles (°)	1.017	2.065	0.916	1.156	1.141
**Ramachandran Plot Statistics**					
In Favoured Regions (%)	292 (99.0)	192 (98.7)	140 (96.6)	516 (96.5)	456 (95.0)
In Allowed Regions (%)	3 (1.0)	2 (1.1%)	5 (3.5)	19 (3.5)	24 (5.0)
Outliers (%)	0 (0)	0 (0)	0 (0)	0 (0)	0 (0)

Values in parentheses are for the highest resolution shell.
