# Supplementary material for: OTU Deubiquitinases Reveal Mechanisms of Linkage Specificity and Enable Ubiquitin Chain Restriction Analysis
Source: Cell. 2013 Jul 3;154(1):169–84. doi: 10.1016/j.cell.2013.05.046 (PMC3705208; doi:10.1016/j.cell.2013.05.046)
Supplement: Data S1. Sequence Alignments of OTUD1, OTUD2, and OTUD3 from Different Species, Related to Figures 5 and 6 — Sequence alignments of OTUD1 (A), OTUD2 (B), and OTUD3 (C) catalytic domains as observed in the crystal structure, derived from the Ensembl database (www.ensembl.org). Secondary structure elements for the human sequence are as indicated, the Cys and His loops are shown in pink, catalytic residues are labeled by stars, and S2 site residues in OTUD2 are shown in green. Black triangles indicate the crystallized constructs. OTUD1 and OTUD2 alignments cover the C-terminal UIM and ZnF sequences, respectively, that were not resolved in the crystal structures. [file mmc2.pdf]

OTUD1 OTU+UIM

```
ENSP00000365678_Hsap_/287-481         290       300       310       320       330       340       350
ENSP00000365678_Hsap_/287-481      DEKLALYLAEVEKQDKYLRQRNKYRFHIIPDGNCLYRAVSKTVYGDQSLHRELREQTVHYIADHLDHFSP
ENSPTRP00000003995_Ptro_/286-480   DEKLALYLAEVEKQDKYLRQRNKYRFHIIPDGNCLYRAVSKTVYGDQSLHRELREQTVHYIADHLDHFSP
ENSGGOP00000000728_Ggor_/287-481   DEKLALYLAEVEKQDKYLRQRNKYRFHIIPDGNCLYRAVSKTVYGDQSLHRDLREQTVHYIADHLDHFSP
ENSMMUP00000012116_Mmul_/286-480   DEKLALYLAEVEKQDKYLRQRNKYRFHIIPDGNCLYRAVSKTVYGDQSLHRELREQTVHYIADHLDHFSP
ENSTGUP00000001214_Tgut_/5-199     SEKLALYLAEVEKQDKYLRQKGRFRFHIIPDGNCLYRAVCKAVYGDQRLHGELREQTVHYIADHLDHFNP
ENSGALP00000030602_Ggal_/7-201     SEKLALYLAEVEKQDRYLRQKGRFRFHIIPDGNCLYRAVCKAVYGDQRPHGELREQTVHYIADHLDHFGP
ENSPSIP00000001551_Psin_/6-200     SEKLALYLAEVEKQDKYLRHKGRFRFHIIPDGNCLYRAVCKTVYGDQRLHSDLREQTVHYIADHLHHFSP
ENSCAFP00000006263_Cfam_/129-323   DEKLALYLAEVERQDKYLRQRSKYRFHIIPDGNCLYRAVSKTVYGDQSLHRELREQTVHYIADHLDHFSP
ENSLAFP00000024360_Lafr_/176-370   DEKLALYLAEVERQDKYLRQRNKYRFHIIPDGNCLYRAVSKTVYGNQSLHRELREQTVHYIADHLDHFSP
ENSOGAP00000015864_Ogar_/210-404   DEKLALYLAEVERQDKYLRQRNKYRFHIIPDGNCLYRAVSKTVYGDQSLHRELREQTVHYIADHLDHFSP
ENSBTAP00000056095_Btau_/268-462   DEKLALYLAEVERQDKYLRQRNKYRFHIIPDGNCLYRAVSKAVYGDQSLHRELREQTVHYIADHLDHFSP
ENSCJAP00000014528_Cjac_/265-459   DEKLALYLAEVEKQDKYLRQRNKYRFHIIPDGNCLYRAVSKTVYGDQSLHRELREQTVHYIADHLDHFNP
ENSXMAP00000020100_Xmac_/5-199     NDKVTRYLAEVERQNKYLQERHKYRYHIIPDGNCLYRAVCKATYGDQARHGELREQTVHHIADHLDEFIP
ENSTRUP00000045767_Trub_/27-221    DDKVTRYLAEVETQNQYLQERRKYRYHIIPDGNCLYRAVCKATYGDQGRHGELREETVHHIADHLDEFNP
ENSGACP00000005044_Gacu_/27-221    NSKVTRYLAEVEKQNKYLQERRKYRYHIIPDGNCLYRAVCKATCGDQAGHRELREQTVHHIADHLEEFNA
ENSOANP00000021049_Oana_/48-242    DERLARHLAEVDKQDRYLRQRSKFRFHIIPDGNCLYRAVSRAVHGDQSLHRDLRERTVHYIADHLDRFGP
ENSTNIP00000015745_Tnig_/30-223    NDKVTRYLAEVEKQNQYLQERHKYRYHIIPDGNCLYRAVCKATYGDQARHGELREETVHHIADHLDQFNP
ENSGMOP00000019561_Gmor_/20-213    RDQVSRYLAEVEEQNAYLRERNKFRFQVIPDGNCLYRAVCRATSGDQSGHLELRERTIHHIADHLEQFSH
ENSMUSP00000100617_Mmus_/260-454   DEKLALYLAEVERQDKYLRQRNKYRFHIIPDGNCLYRAVSKTVYGDQSLHRELREQTVHYIADHLDHFSP
ENSRNOP00000060310_Rnor_/261-455   DEKLALYLAEVERQDKYLRQRNKYRFHIIPDGNCLYRAVSKTVYGDQSLHRELREQTVHYIADHLDHFSP
ENSMODP00000011112_Mdom_/201-395   DEKLALYLAEVEKQDKYLRQRSKYRFHIIPDGNCLYRAVSKAMYGDQSLHPELREQTVHYIADHLDHFGP
ENSLACP00000013142_Lcha_/239-433   NEKIARYLAEVDKQNKYLRDRQKYRFHIIPDGNCLYRAVSKAVYGDQAMHKDLREQTVHHIADHLDEFNP
```

```
ENSP00000365678_Hsap_/287-481        360       370       380       390       400       410          420
ENSP00000365678_Hsap_/287-481      LIEGDVGEFIIAAAQDGAWAGYPELLAMGQMLNVNIHLTTGGRLESPTVSTMIHYLG...PEDSLRPSIW
ENSPTRP00000003995_Ptro_/286-480   LIEGDVGEFIIAAAQDGAWAGYPELLAMGQMLNVNIHLTTGGRLESPTVSTMIHYLG...PEDSLRPSIW
ENSGGOP00000000728_Ggor_/287-481   LIEGDVGEFIIAAAQDGAWAGYPELLAMGQMLNVNIHLTTGGRLESPTVSTMIHYLG...PEDSLRPSIW
ENSMMUP00000012116_Mmul_/286-480   LIEGDVGEFIIAAAQDGAWAGYPELLAMGQMLNVNIHLTTGGRLESPTVSTMIHYLG...PEDSLRPSIW
ENSTGUP00000001214_Tgut_/5-199     IIEGDVGEFLIGAAQDGAWAGYPELLAMGQMLNVNIHLTTGGRPESPTVSTMVHYLG...PEDPTRPSIW
ENSGALP00000030602_Ggal_/7-201     IIEGDVGEFLIAAAQDGAWAGYPELLAMGQMLNVNIHLTTGGRPESPTVSTMVHYLG...PEDPTRPSIW
ENSPSIP00000001551_Psin_/6-200     IIEGDVGEFIIGAAQDGAWAGYPELLAMGQMLNINIHLTTGGRPESPTVSTMAHYLG...PEDPSRPSIW
ENSCAFP00000006263_Cfam_/129-323   LIEGDVGEFIIAAAQDGAWAGYPELLAMGQMLNVNIHLTTGGRLESPTVSTMIHYLG...PEDSLRPSIW
ENSLAFP00000024360_Lafr_/176-370   LIEGDVGEFLIAAAQDGAWAGYPELLAMGQMLNVNIHLTTGGRLESPTVSTMIHYLG...PEDSLRPSIW
ENSOGAP00000015864_Ogar_/210-404   LIEGDVGEFIIAAAQDGAWAGYPELLAMGQMLNVNIHLTTGGRLESPTVSTMIHYLG...PEDSLRPSIW
ENSBTAP00000056095_Btau_/268-462   LIEGDVGEFIIAAAQDGAWAGYPELLAMGQMLNVNIHLTTGGRLESPTVSTMIHYLG...PEDSLRPSIW
ENSCJAP00000014528_Cjac_/265-459   LIEGDVGEFLIAAAQDGAWAGYPELLAMGQMLNVNIHLTTGGRLESPTVSTMIHYLG...PEDSLRPSIW
ENSXMAP00000020100_Xmac_/5-199     IIEGDVGEFLISAAQDGAWAGYPELLAMSQMLNVNIHLTTGGSLESFTVSTMVHYLG...EEDASKPAIW
ENSTRUP00000045767_Trub_/27-221    IIEGDVGEFLISAAQDGTWAGYPELLAMSQMLNTTVYLTTGGSLESPTVSTMGHYLG...EDDPSRPAIW
ENSGACP00000005044_Gacu_/27-221    IIEGDVGEFLINAAQDGAWAGYPELHAMSQMLSVDVHLTTGGSSESPTVSTMVHYLG...QEDASKGAVW
ENSOANP00000021049_Oana_/48-242    LIEGDVGEFLVAAAQDGAWAGYPELLAMGQMLDVNILLTTGGRAESPTVATMSHYLG...PDDGRRPSIW
ENSTNIP00000015745_Tnig_/30-223    ILEGDVGEFLINAAQDGA.AGYPELLAMSQMLNTTIYLTTGGSLESPTVSTMGHYLG...QDDPSRPAIW
ENSGMOP00000019561_Gmor_/20-213    VIEGDVGEFLISASQDGAWAGYPELLAMSQMLQVHVHLTTGGGARSPTVSTMVHYLGDEHENAPADDIW
ENSMUSP00000100617_Mmus_/260-454   LIEGDVGEFIIAAAQDGAWAGYPELLAMGQMLNVNIHLTTGGRLESPTVSTMIHYLG...PEDSLRPSIW
ENSRNOP00000060310_Rnor_/261-455   LIEGDVGEFIIAAAQDGAWAGYPELLAMGQMLNVNIHLTTGGRLESPTVSTMVHYLG...PEDSLRPSIW
ENSMODP00000011112_Mdom_/201-395   LIEGDVGEFIIAAAQDGSWAGYPELLAMGQMLNVNIHLTTGGRLESPTVSTMIHYLG...PEDSLRPSIW
ENSLACP00000013142_Lcha_/239-433   IIEGDIGEFLINAAQDGTWAGYPELLAMSQMLNINIYLTTGGSLESPTVSTMVHYLG...AEDAMKTSIW
```

```
ENSP00000365678_Hsap_/287-481           430       440       450       460       470       480
ENSP00000365678_Hsap_/287-481      LSWLSNGHYDAVFDHSYPNPEYDNWCKQTQVQRKRDEELAKSMAISLSKMYIEQNACS
ENSPTRP00000003995_Ptro_/286-480   LSWLSNGHYDAVFDHSYPNPEYDNWCKQTQVQRKRDEELAKSMAISLSKMYIEQNACS
ENSGGOP00000000728_Ggor_/287-481   LSWLSNGHYDAVFDHSYPNPEYDNWCKQTQVQRKRDEELAKSMAISLSKMYIEQNACS
ENSMMUP00000012116_Mmul_/286-480   LSWLSNGHYDAVFDHSYPNPEYDNWCKQTQVQRKRDEELAKSMAISLSKMYIEQNACS
ENSTGUP00000001214_Tgut_/5-199     LSWLSNGHYDAVLDRVCPNPEYEAWCRQTQVQRRRDEELAKSMAVSLSKMYIEQNACS
ENSGALP00000030602_Ggal_/7-201     LSWLSNGHYDAVLDRVCPNPEYEAWCRQTQVQRRRDEELAKSMAVSLSKMYIEQNACS
ENSPSIP00000001551_Psin_/6-200     LSWLSNGHYDAVLDCECPNPEYEAWCRQTQVQRRRDEELAKSMAMSLSKMYIEQNTCS
ENSCAFP00000006263_Cfam_/129-323   LSWLSNGHYDAVFDHSYPNPEYDNWCRQTQVQRKRDEELAKSMAISLSKMYIEQNACS
ENSLAFP00000024360_Lafr_/176-370   LSWLSNGHYDAVFDHSYPNPEYDNWCKQTQVQRKRDEELAKSMAISLSKMYIEQNACS
ENSOGAP00000015864_Ogar_/210-404   LSWLSNGHYDAVFDHCYPNPEYDNWCKQTQVQRKRDEELAKSMAISLSKMYIEQNACS
ENSBTAP00000056095_Btau_/268-462   LSWLSNGHYDAVFDHSYPNPEYDTWCRQTQVQRKRDEELAKSMAISLSKMYIEQNACS
ENSCJAP00000014528_Cjac_/265-459   LSWLSNGHYDAVFDHSYPNPEYDNWCKQTQVQRKRDEELAKSMAISLSKMYIEQNACS
ENSXMAP00000020100_Xmac_/5-199     LSWLSNGHYDVVLDKRLPNPEYEDWCRQSQMQRKRDEDLAKSMAASLSKMYLEQNGYV
ENSTRUP00000045767_Trub_/27-221    LSWLSNGHYDVLLDKSLPNPEYEDWCRHSQMQRKRDEELAKSMAASLSKMYSEQNGCA
ENSGACP00000005044_Gacu_/27-221    LSWLSNGHYDALLDECLPNPEYEDWCSRSQMQRQRDEELAKSMAASLSRMYIEQNGSA
ENSOANP00000021049_Oana_/48-242    LSWLSNGHYDAVFDRCHPNPEYDSWCRQTQVQRKRDEELAKSMAVSLSKMYIEQNTCS
ENSTNIP00000015745_Tnig_/30-223    LSWLSNGHYDVLLDKSLPNPEYEDWCRHSQMQKRRDEELAKSMAASLSKMYIEQNGVC
ENSGMOP00000019561_Gmor_/20-213    LSWLSNGHYDVILDE.LPNPEYEDWCMQAQAQRRRDEELARAMAASLSRMYLEQN...
ENSMUSP00000100617_Mmus_/260-454   LSWLSNGHYDAVFDHSYPNPEYDNWCKQTQIQKKRDEELAKSMAISLSKMYIEQNACS
ENSRNOP00000060310_Rnor_/261-455   LSWLSNGHYDAVFDHSYPNPEYDNWCKQTQMQRKRDEELAKSMAISLSKMYIEQNACS
ENSMODP00000011112_Mdom_/201-395   LSWLSNGHYDAVFDHSYPNPEYDNWCKQTQVQRKRDEELARSMAISLSKMYIEQNACS
ENSLACP00000013142_Lcha_/239-433   LSWLSNGHYDAVFDHPVLNPEYESWCKQTQVQRKRDEELAKSMAASLSKMYIEQNGYS
```

OTUD2 OTU+ZnF

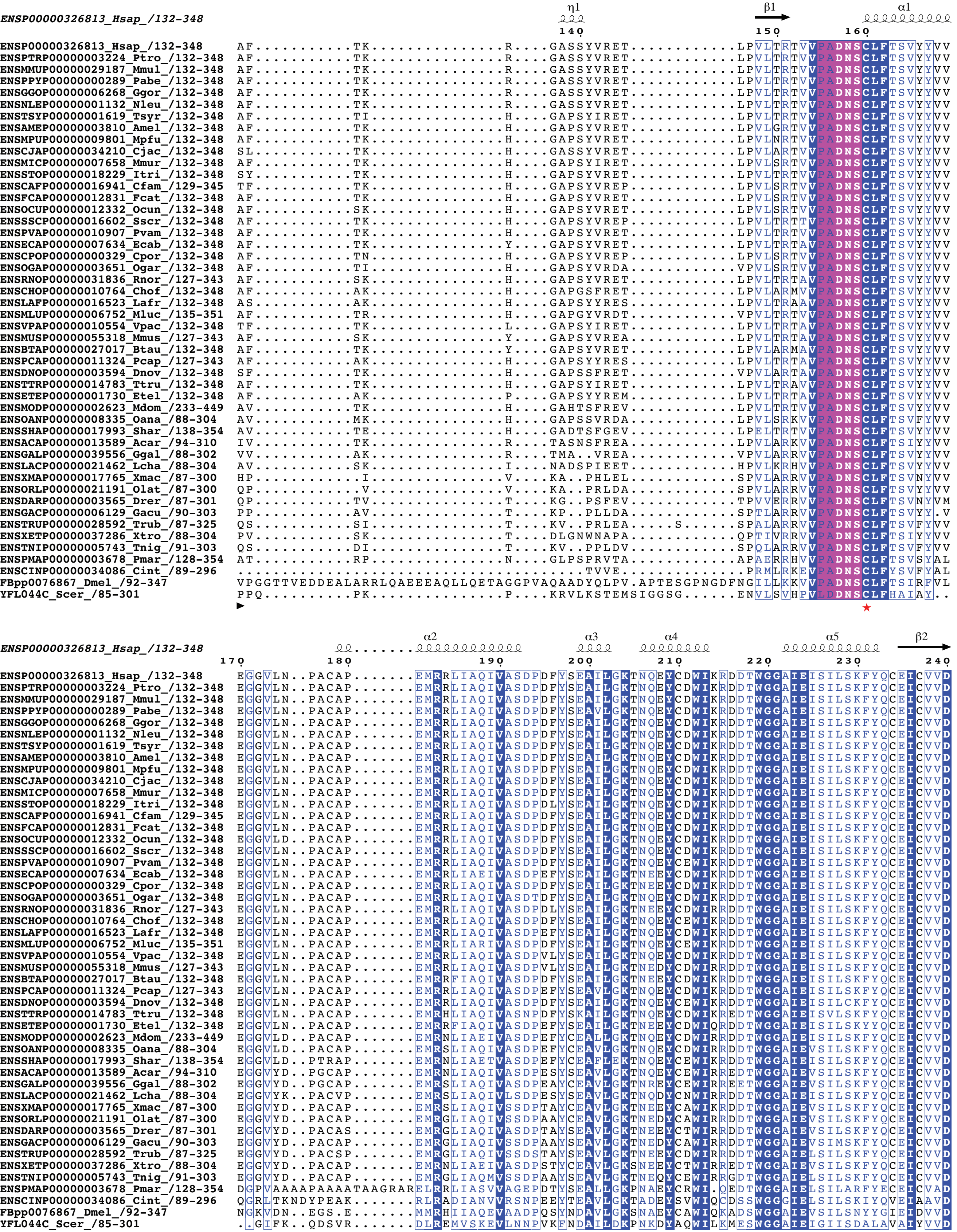

OTUD2 OTU+ZnF

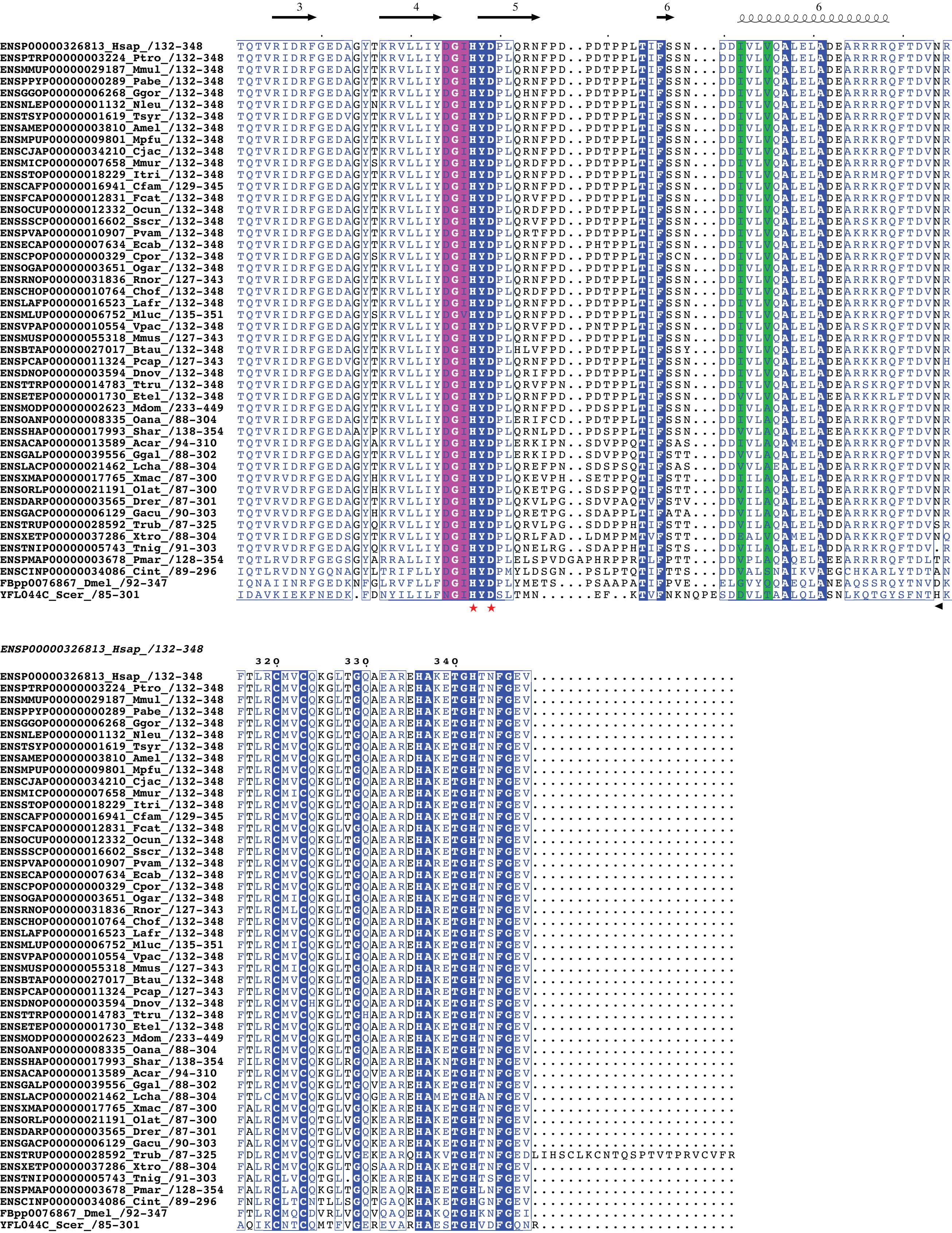

OTUD3 OTU

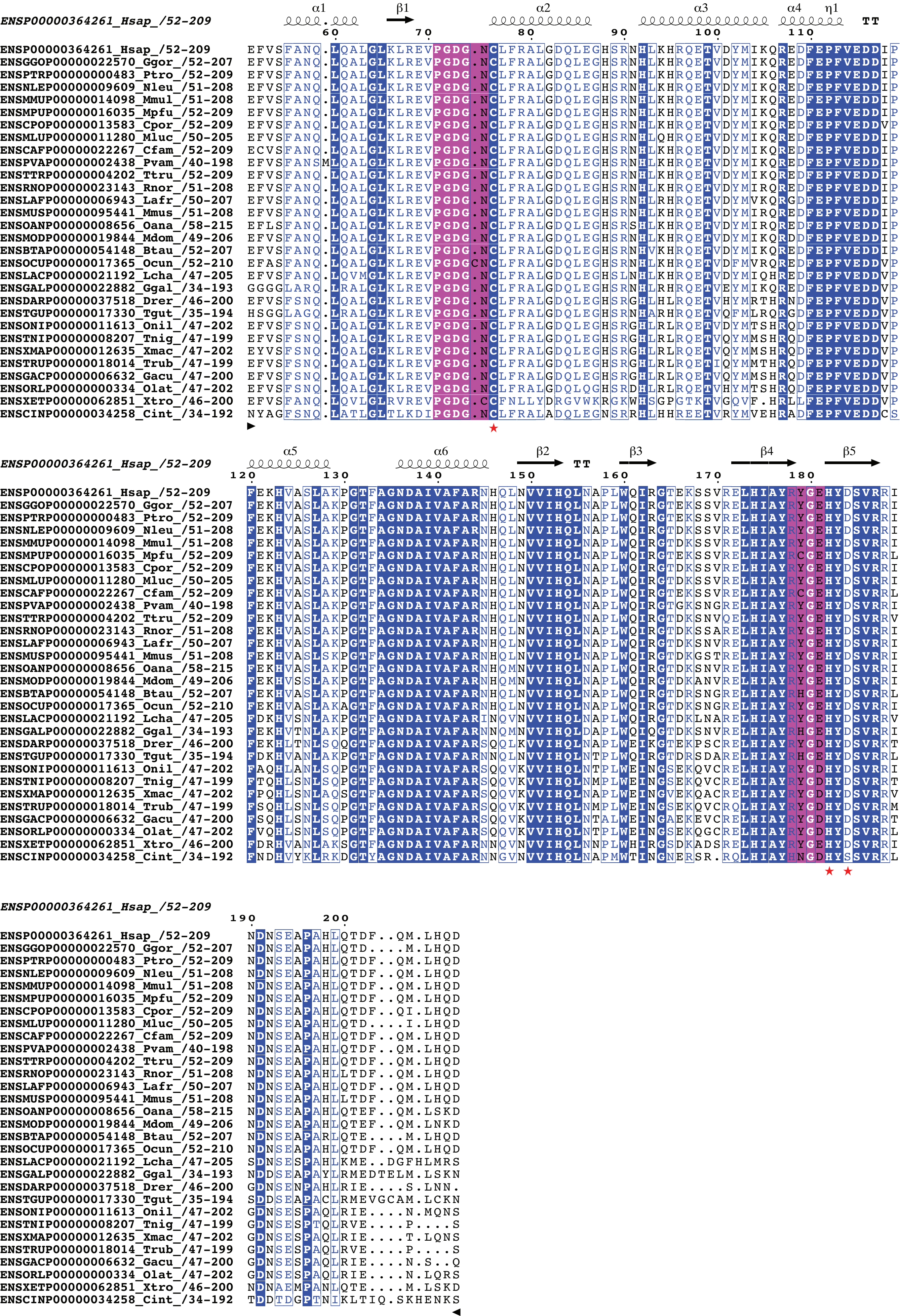

ENSP00000364261_Hsap_/52-209
α1 β1 α2 α3 α4 η1 TT
60 70 80 90 100 110
ENSP00000364261_Hsap_/52-209 EFVSFANQ.LQALGLKLREVPGDG.NCLFRALGDQLEGHSRNHLKHRQETVDYMIKQREDFEPFVEDDIP
ENSGGOP00000022570_Ggor_/52-207 EFVSFANQ.LQALGLKLREVPGDG.NCLFRALGDQLEGHSRNHLKHRQETVDYMIKQREDFEPFVEDDIP
ENSPTRP00000000483_Ptro_/52-209 EFVSFANQ.LQALGLKLREVPGDG.NCLFRALGDQLEGHSRNHLKHRQETVDYMIKQREDFEPFVEDDIP
ENSNLEP00000009609_Nleu_/51-208 EFVSFANQ.LQALGLKLREVPGDG.NCLFRALGDQLEGHSRNHLKHRQETVDYMIKQREDFEPFVEDDIP
ENSMMUP00000014098_Mmul_/51-208 EFVSFANQ.LQALGLKLREVPGDG.NCLFRALGDQLEGHSRNHLKHRQETVDYMIKQREDFEPFVEDDIP
ENSMPUP00000016035_Mpfu_/52-209 EFVSFANQ.LQALGLKLREVPGDG.NCLFRALGDQLEGHSRNHLKHRQETVDYMIKQREDFEPFVEDDIP
ENSCPOP00000013583_Cpor_/52-209 EFVSFANQ.LQALGLKLREVPGDG.NCLFRALGDQLEGHSRNHLKHRQETVDYMIKQREDFEPFVEDDVP
ENSMLUP00000011280_Mluc_/50-205 EFVSFANQ.LQALGLKLREVPGDG.NCLFRALGDQLEGHSRNHLQHRQETVDYMIKQREDFEPFVEDDIP
ENSCAFP00000022267_Cfam_/52-209 ECVSFANQ.LQALGLKLREVPGDG.NCLFRALGDQLEGHSRNHLKHRQETVDYMIKQREDFEPFVEDDIP
ENSPVAP00000002438_Pvam_/40-198 EFVSFANSMLQALGLKLREVPGDG.NCLFRALGDQLEGHSRNHLKHRQETVDYMIKQREDFEPFVEDDIP
ENSTTRP00000004202_Ttru_/52-209 EFVSFANQ.LQALGLKLREVPGDG.NCLFRALGDQLEGHSRNHLKHRQETVDYMIKQREDFEPFVEDDIP
ENSRNOP00000023143_Rnor_/51-208 EFVSFANQ.LQALGLKLREVPGDG.NCLFRALGDQLEGHSRNHLKHRQETVDYMIRQREDFEPFVEDDIP
ENSLAFP00000006943_Lafr_/50-207 EFVSFANQ.LQALGLKLREVPGDG.NCLFRALGDQLEGHSRNHLRHRQETVDYMVKQRGDFEPFVEDDIP
ENSMUSP00000095441_Mmus_/51-208 EFVSFANQ.LQALGLKLREVPGDG.NCLFRALGDQLEGHSRNHLKHRQETVDYMIRQREDFEPFVEDDIP
ENSOANP00000008656_Oana_/58-215 EFLSFANQ.LQALGLKLREVPGDG.NCLFRALGDQLEGHSRSHLKHRQETVDYMIRQREDFEPFVEDDIP
ENSMODP00000019844_Mdom_/49-206 EFVSFANQ.LQALGLKLREVPGDG.NCLFRALGDQLEGHSRNHLKHRQETVDYMIKQRDDFEPFVEDDIP
ENSBTAP00000054148_Btau_/52-207 EFVSFANQ.LQALGLKLREVPGDG.NCLFRALGDQLEGHSRNHVKHRQETVDYMIKQREDFEPFVEDDIP
ENSOCUP00000017365_Ocun_/52-210 EFASFANQ.LQALGLKLREVPGDGCNCLFRALGDQLEGHSRNHLKHRQETVDFMVRQREDFEPFVEDDVP
ENSLACP00000021192_Lcha_/47-205 EFVSFANQ.LQVMGLKLREVPGDG.NCLFRALGDQLEGHSLNHLKHRQETVDYMIQHREDFEPFVEDDVP
ENSGALP00000022882_Ggal_/34-193 GGGGLARQ.LRALGLKLREVPGDG.NCLFRALGDQLEGHSRNHLRHRQETVEYMIKQREDFEPFVEDDVP
ENSDARP00000037518_Drer_/46-200 EFVSFSNQ.LQALGLKLREVPGDG.NCLFRALGDQLEGHSRGHLHLRQETVHYMRTHRNDFEPFVEDDVP
ENSTGUP00000017330_Tgut_/35-194 HSGGLAGQ.LRALGLKLREVPGDG.NCLFRALGDQLEGHSRNHARHRQETVQFMLRQRGDFEPFVEDDVP
ENSONIP00000011613_Onil_/47-202 EFVSFSNQ.LQALGLKLREVPGDG.NCLFRALGDQLEGHSRGHLRLRQETVDYMTSHRQDFEPFVEDDVP
ENSTNIP00000008207_Tnig_/47-199 EFVSFSNQ.LQALGLKLREVPGDG.NCLFRALGDQLEGHSRGHLRLRQETVQYMMSHRQDFEPFVEDDVP
ENSXMAP00000012635_Xmac_/47-202 EYVSFSNQ.LQALGLKLREVPGDG.NCLFRALGDQLEGHSRGHLRLRQETVQYMMSHRQDFEPFVEDDVP
ENSTRUP00000018014_Trub_/47-199 EFVSFSNQ.LQALGLKLREVPGDG.NCLFRALGDQLEGHSRGHLRLRQETIQYMMTHRQDFEPFVEDDVP
ENSGACP00000006632_Gacu_/47-200 EFVSFSNQ.LQALGLKLREVPGDG.NCLFRALGDQLEGHSRGHLRLRQETVQYMMSHRQDFEPFVEDDVP
ENSORLP00000000334_Olat_/47-202 EFVSFSNQ.LQALGLKLREVPGDG.NCLFRALGDQLQGHSRDHLRLRQETVHYMTSHRQDFEPFVEDDVP
ENSXETP00000062851_Xtro_/46-200 EFVSFANQ.LQVLGLRVREVPGDG.CCFNLLYDRGVWKRGKWHSGPGTKTVGQVF.HRLLFEPFVEDDVP
ENSCINP00000034258_Cint_/34-192 NYAGFSNQ.LATLGLTLKDIPGDG.NCLFRALADQLEGNSRRHLHHREETVRYMVEHRADFEPFVEDDCS
ENSP00000364261_Hsap_/52-209
α5 α6 β2 TT β3 β4 β5
120 130 140 150 160 170 180
ENSP00000364261_Hsap_/52-209 FEKHVASLAKPGTFAGNDAIVAFARNHQLNVVIHQLNAPLWQIRGTEKSSVRELHIAYRYGEHYDSVRRI
ENSGGOP00000022570_Ggor_/52-207 FEKHVASLAKPGTFAGNDAIVAFARNHQLNVVIHQLNAPLWQIRGTEKSSVRELHIAYRYGEHYDSVRRI
ENSPTRP00000000483_Ptro_/52-209 FEKHVASLAKPGTFAGNDAIVAFARNHQLNVVIHQLNAPLWQIRGTEKSSVRELHIAYRYGEHYDSVRRI
ENSNLEP00000009609_Nleu_/51-208 FEKHVASLAKPGTFAGNDAIVAFARNHQLNVVIHQLNAPLWQIRGTDKSSVRELHIAYRYGEHYDSVRRI
ENSMMUP00000014098_Mmul_/51-208 FEKHVASLAKPGTFAGNDAIVAFARNHQLNVVIHQLNAPLWQIRGTDKSSVRELHIAYRYGEHYDSVRRI
ENSMPUP00000016035_Mpfu_/52-209 FEKHVASLAKPGTFAGNDAIVAFARNHQLNVVIHQLNAPLWQIRGTEKSSVRELHIAYRCGEHYDSVRRL
ENSCPOP00000013583_Cpor_/52-209 FEKHVASLAKPGTFAGNDAIVAFARNHQLNVVIHQLNAPLWQIRGTDKSSVRELHIAYRYGEHYDSVRRI
ENSMLUP00000011280_Mluc_/50-205 FEKHVASLAKPGTFAGNDAIVAFARNHQLNVVIHQLNAPLWQIRGTDKSSVRELHIAYRYGEHYDSVRRI
ENSCAFP00000022267_Cfam_/52-209 FEKHVASLAKPGTFAGNDAIVAFARNHQLNVVIHQLNAPLWQIRGTEKSSVRELHIAYRCGEHYDSVRRL
ENSPVAP00000002438_Pvam_/40-198 FEKHVASLAKPGTFAGNDAIVAFARNHQLNVVIHQLNAPLWQIRGTGKSNGRELHIAYRYGEHYDSVRRI
ENSTTRP00000004202_Ttru_/52-209 FEKHVASLAKPGTFAGNDAIVAFARNHQLNVVIHQLNAPLWQIRGTDKSNVQELHIAYRYGEHYDSVRRV
ENSRNOP00000023143_Rnor_/51-208 FEKHVASLAKPGTFAGNDAIVAFARNHQLNVVIHQLNAPLWQIRGTDKSSARELHIAYRYGEHYDSVRRI
ENSLAFP00000006943_Lafr_/50-207 FEKHVASLAKPGTFAGNDAIVAFARNHQLNVVIHQLNAPLWQIRGTDKSNVRELHIAYRYGEHYDSVRRI
ENSMUSP00000095441_Mmus_/51-208 FEKHVASLSKPGTFAGNDAIVAFARNHQLNVVIHQLNAPLWQIRGTDKGSTRELHIAYRYGEHYDSVRRI
ENSOANP00000008656_Oana_/58-215 FEKHVASLAKPGTFAGNDAIVAFARNHQMNVVIHQLNAPLWQIRGTDKSNVRELHIAYRYGEHYDSVRRI
ENSMODP00000019844_Mdom_/49-206 FEKHVSSLAKPGTFAGNDAIVAFARNHQMNVVIHQLNAPLWQIRGTDKANVRELHIAYRYGEHYDSVRRI
ENSBTAP00000054148_Btau_/52-207 FEKHVASLAKPGTFAGNDAIVAFARNHQLNVVIHQLNAPLWQIRGTDRSNGRELHIAYRHGEHYDSVRRL
ENSOCUP00000017365_Ocun_/52-210 FEKHVASLAKAGTFAGNDAIVAFARNHQLNVVIHQLNAPLWQIRGTDKSNGRELHIAYRYGEHYDSVRRI
ENSLACP00000021192_Lcha_/47-205 FDKHVSNLAKPGTFAGNDAIVAFARINQVNVVIHQLNAPLWQIRGTDKLNARELHIAYRYGEHYDSVRRV
ENSGALP00000022882_Ggal_/34-193 FEKHVTNLAKPGTFAGNDAIVAFARNHQMNVVIHQLDAPLWQIQGTDKSNVRELHIAYRHGEHYDSVRRI
ENSDARP00000037518_Drer_/46-200 FEKHLTNLSQQGTFAGNDAIVAFARSQQLKVVIHQLNAPLWEIKGTEKPSCRELHIAYRYGDHYDSVRKT
ENSTGUP00000017330_Tgut_/35-194 FDKHVANLAKPGTFAGNDAIVAFARNNQLNVVIHQLNTPLWQIRGTDRSDARELHIAYRHGEHYDSVRRL
ENSONIP00000011613_Onil_/47-202 FAQHLANLSQPGTFAGNDAIVAFARSQQVKVVIHQLNTPLWEINGSEKQVCRELHIAYRYGDHYDSVRRI
ENSTNIP00000008207_Tnig_/47-199 FTQHLSNLSQPGTFAGNDAIVAFARSQQVKVVIHQLNMPLWEINGSEKQVCRELHIAYRYGDHYDSVRRT
ENSXMAP00000012635_Xmac_/47-202 FPQHLSNLAQSGTFAGNDAIVAFARSQQVKVVIHQLNAPLWEINGVEKQACRELHIAYRYGDHYDSVRRV
ENSTRUP00000018014_Trub_/47-199 FSQHLSNLSQPGTFAGNDAIVAFARSQQVKVVIHQLNMPLWEINGSEKQVCRQLHIAYRYGDHYDSVRRM
ENSGACP00000006632_Gacu_/47-200 FSQHLSNLSQPGTFAGNDAIVAFARSQQVKVVIHQLNTALWEINGAEKEVCRELHIAYRYGDHYDSVRRT
ENSORLP00000000334_Olat_/47-202 FVQHLSNLSQPGTFAGNDAIVAFARSQQVKVVIHQLNTPLWEINGSEKQGCRELHIAYRYGDHYDSVRRL
ENSXETP00000062851_Xtro_/46-200 FDRHVANLAKSGTFAGNDAIVAFARNNQVNVVIHQLNNPLWHIRGSDKADSRELHIAYRYGDHYDSVRRI
ENSCINP00000034258_Cint_/34-192 FNDHVYKLRKDGTYAGNDAIVAFARNNGVNVVIHQLNAPMWTINGNERSR.RQLHIAYHNGDHYSSVRKL
ENSP00000364261_Hsap_/52-209
190 200
ENSP00000364261_Hsap_/52-209 NDNSEAPAHLQTDF..QM.LHQD
ENSGGOP00000022570_Ggor_/52-207 NDNSEAPAHLQTD....M.LHQD
ENSPTRP00000000483_Ptro_/52-209 NDNSEAPAHLQTDF..QM.LHQD
ENSNLEP00000009609_Nleu_/51-208 NDNSEAPAHLQTDF..QM.LHQD
ENSMMUP00000014098_Mmul_/51-208 NDNSEAPAHLQTDF..QM.LHQD
ENSMPUP00000016035_Mpfu_/52-209 NDNSEAPAHLQTDF..QM.LHQD
ENSCPOP00000013583_Cpor_/52-209 NDNSEAPAHLQTDF..QI.LHQD
ENSMLUP00000011280_Mluc_/50-205 NDNSEAPAHLQTD....I.LHQD
ENSCAFP00000022267_Cfam_/52-209 NDNSEAPAHLQTDF..QM.LHQD
ENSPVAP00000002438_Pvam_/40-198 NDNSEAPAHLQTDF..QM.LHQD
ENSTTRP00000004202_Ttru_/52-209 NDNSEAPAHLQTDF..QM.LHQD
ENSRNOP00000023143_Rnor_/51-208 NDNSEAPAHLLTDF..QM.LHQD
ENSLAFP00000006943_Lafr_/50-207 NDNSEAPAHLQTDF..QM.LHQD
ENSMUSP00000095441_Mmus_/51-208 NDNSEAPAHLLTDF..QM.LHQD
ENSOANP00000008656_Oana_/58-215 NDNSEAPAHLQTEF..QM.LSKD
ENSMODP00000019844_Mdom_/49-206 NDNSEAPAHLQTEF..QM.LNKD
ENSBTAP00000054148_Btau_/52-207 NDNSEAPARLQTE....M.LHQD
ENSOCUP00000017365_Ocun_/52-210 NDNSEAPAHLQTDF..QM.LHQD
ENSLACP00000021192_Lcha_/47-205 SDNSESPAHLKME..DGFHLMRS
ENSGALP00000022882_Ggal_/34-193 NDDSEAPAYLRMEDTELM.LSKN
ENSDARP00000037518_Drer_/46-200 GDNSENPAHLRIE....S.LNN.
ENSTGUP00000017330_Tgut_/35-194 SDDSEAPACLRMEVGCAM.LCKN
ENSONIP00000011613_Onil_/47-202 GDNSESPAQLRIE....N.MQNS
ENSTNIP00000008207_Tnig_/47-199 GDNSESPTQLRVE....P....S
ENSXMAP00000012635_Xmac_/47-202 GDNSESPAQLRIE....T.LQNS
ENSTRUP00000018014_Trub_/47-199 GDNSESPAQLRVE....P....S
ENSGACP00000006632_Gacu_/47-200 GDNSESPAQLRIE....N.S..Q
ENSORLP00000000334_Olat_/47-202 GDNSESPAQLRIE....N.LQRS
ENSXETP00000062851_Xtro_/46-200 NDNAEMPANLQTE....M.LSKD
ENSCINP00000034258_Cint_/34-192 TDDTDGPTNIKLTIQ.SKHENKS
