# Supplementary material for: OTU Deubiquitinases Reveal Mechanisms of Linkage Specificity and Enable Ubiquitin Chain Restriction Analysis
Source: Cell. 2013 Jul 3;154(1):169–84. doi: 10.1016/j.cell.2013.05.046 (PMC3705208; doi:10.1016/j.cell.2013.05.046)
Supplement: Document S1. Article plus Supplemental Information [file mmc3.pdf]

Cell

OTU Deubiquitinases Reveal Mechanisms of Linkage Specificity and Enable Ubiquitin Chain Restriction Analysis

Tycho E.T. Mevissen,[1,2] Manuela K. Hospenthal,[1,6] Paul P. Geurink,[3,6] Paul R. Elliott,[1,6] Masato Akutsu,[1,4] Nadia Arnaudo,[1] Reggy Ekkebus,[3] Yogesh Kulathu,[1,5] Tobias Wauer,[1] Farid El Oualid,[3] Stefan M.V. Freund,[1] Huib Ovaa,[3] and David Komander[1,*]
[1]Medical Research Council Laboratory of Molecular Biology, Francis Crick Avenue, Cambridge Biomedical Campus, Cambridge CB2 0QH, UK
[2]Freie Universität Berlin, Fachbereich Biologie, Chemie, Pharmazie, D-14195 Berlin, Germany
[3]Division of Cell Biology, Netherlands Cancer Institute, Plesmanlaan 121, 1066 CX Amsterdam, the Netherlands
[4]Present address: Buchmann Institute for Molecular Life Sciences, Goethe University Frankfurt, Max-von-Laue- Straße 15, 60438 Frankfurt am Main, Germany
[5]Present address: Medical Research Council Protein Phosphorylation and Ubiquitination Unit, Dow Street, Dundee DD15EH, UK
[6]These authors contributed equally to this work
*Correspondence: dk@mrc-lmb.cam.ac.uk
http://dx.doi.org/10.1016/j.cell.2013.05.046
This is an open-access article distributed under the terms of the Creative Commons Attribution License, which permits unrestricted use, distribution, and reproduction in any medium, provided the original author and source are credited.

SUMMARY

Sixteen ovarian tumor (OTU) family deubiquitinases (DUBs) exist in humans, and most members regulate cell-signaling cascades. Several OTU DUBs were reported to be ubiquitin (Ub) chain linkage specific, but comprehensive analyses are missing, and the underlying mechanisms of linkage specificity are unclear. Using Ub chains of all eight linkage types, we reveal that most human OTU enzymes are linkage specific, preferring one, two, or a defined subset of linkage types, including unstudied atypical Ub chains. Biochemical analysis and five crystal structures of OTU DUBs with or without Ub substrates reveal four mechanisms of linkage specificity. Additional Ub-binding domains, the ubiquitinated sequence in the substrate, and defined S1' and S2 Ub-binding sites on the OTU domain enable OTU DUBs to distinguish linkage types. We introduce Ub chain restriction analysis, in which OTU DUBs are used as restriction enzymes to reveal linkage type and the relative abundance of Ub chains on substrates.

INTRODUCTION

Protein ubiquitination is a posttranslational modification of mostly Lys residues that regulates many cellular processes, including protein degradation, intracellular trafficking, cell signaling, autophagy, transcription, translation, and the DNA damage response (Komander and Rape, 2012). This functional diversity is achieved by the ability of ubiquitin (Ub) to form topologically distinct signals. Proteins can be monoubiquitinated at one or multiple sites or polyubiquitinated by modification with Ub chains. Within Ub chains, linkages can be formed via seven Ub Lys residues or via the N-terminal Met1, generating homotypic (one linkage type per polymer) or heterotypic (multiple linkage types per polymer) Ub chains (Komander and Rape, 2012). Differently linked Ub polymers have distinct cellular functions. Lys48-linked Ub chains serve as a proteasomal degradation signal (Hershko and Ciechanover, 1998), whereas Lys63-linked chains are nondegradative and, for example, activate protein kinase cascades (Chen and Sun, 2009). Lys11 linkages constitute an alternative degradation signal used during cell-cycle progression (Wickliffe et al., 2011). Met1-linked chains cooperate with Lys63 linkages in NF-κB signaling (Iwai, 2011). For the remaining four Ub chain types (Lys6, Lys27, Lys29, and Lys33), cellular roles are elusive (Kulathu and Komander, 2012).

Deubiquitinases (DUBs) remove Ub modifications and regulate virtually all Ub-dependent processes (Komander et al., 2009; Reyes-Turcu et al., 2009). Many of the ~80 DUBs that are predicted to be active in human cells have been implicated in human diseases such as neurodegeneration, inflammation, infection, and cancer (Clague et al., 2012). The subfamily of ovarian tumor (OTU) DUBs have emerged as regulators of important signaling cascades. A20 (Hymowitz and Wertz, 2010), OTUD7B/Cezanne (Hu et al., 2013) and OTULIN (Keusekotten et al., 2013) regulate NF-κB signaling, OTUD5/DUBA regulates interferon signaling (Kayagaki et al., 2007), OTUD2/YOD1 and VCPIP regulate p97-mediated processes (Ernst et al., 2009; Wang et al., 2004), and OTUB1 is involved in the DNA damage response (Nakada et al., 2010).

Because of the complexity of the Ub modification, DUBs must display various layers of specificity—they must distinguish not only between Ub and Ub-like modifications but also between

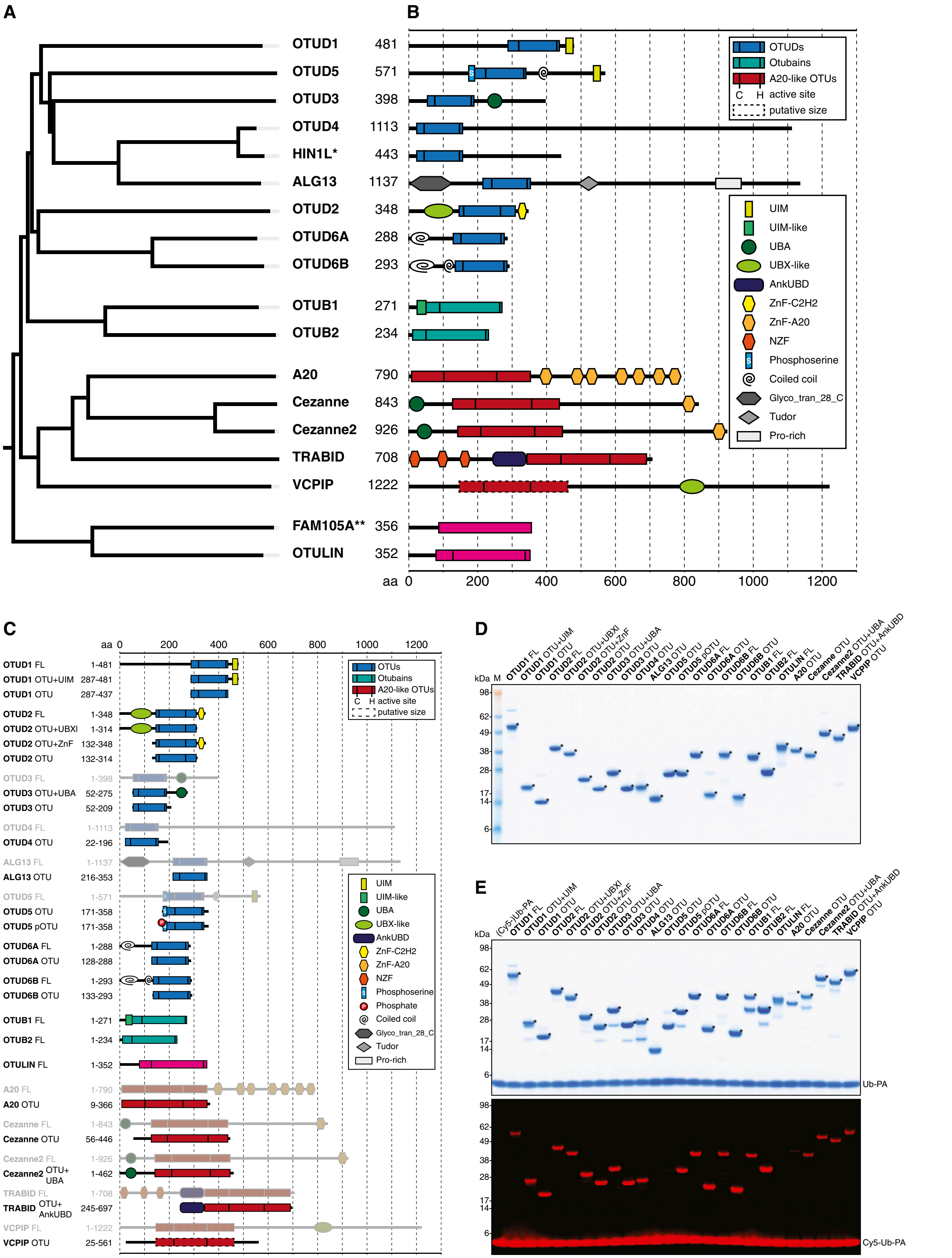

(legend on next page)

the eight Ub linkage types. Moreover, chain topology and length may also affect DUB activity (Komander et al., 2009).

The extent to which DUBs are linkage specific is not clear. Characterized Ub-specific protease (USP) family DUBs are not linkage specific (Faesen et al., 2011). In contrast, OTU family DUBs can be linkage specific. OTUB1 prefers Lys48 linkages (Edelmann et al., 2009; Wang et al., 2009), Cezanne prefers Lys11 linkages (Bremm et al., 2010), TRABID is Lys29 and Lys33 specific (Licchesi et al., 2012), and OTULIN is Met1 specific (Keusekotten et al., 2013). However, with the exception of TRABID and OTULIN, comprehensive analyses comparing all chain types have not been performed.

Here, we provide a biochemical characterization of all 16 human OTU DUBs that contain a complete catalytic triad and analyze their cross-reactivity against Ub-like molecules, catalytic activity, and linkage specificity. Most OTU DUBs show intrinsic linkage specificity, preferring one or a small defined subset of Ub linkage types. Mechanistic and structural studies of three closely related, unstudied OTUs with distinct cleavage profiles revealed four mechanisms for achieving linkage specificity, namely (1) the use of additional Ub-binding domains (UBDs), (2) specific recognition of a ubiquitinated sequence, (3) the use of a conserved S1' Ub-binding site on the OTU domain itself, and (4) the use of an S2 site enabling DUBs to bind longer chains in a linkage-specific manner. The linkage specificity in OTU DUBs can be exploited in Ub chain restriction analysis, whereby linkage-specific DUBs are used to identify the linkage type(s) on a ubiquitinated protein.

RESULTS

The Human OTU Enzymes

In the human genome, OTU domains exist in at least 18 genes, 14 of which have been annotated as active DUBs (Komander et al., 2009). In addition to these, OTULIN/FAM105B (Keusekotten et al., 2013) and ALG13 (UniProt Q9NP73) have recently been described or annotated as additional OTU domains with a complete catalytic triad. FAM105A (UniProt Q9NUU6) contains an OTULIN-like OTU domain but lacks catalytic triad residues. HIN1L is a pseudogene (http://www.ncbi.nlm.nih.gov/gene/360227). Phylogenetic analysis delineates four subfamilies: the OTUB subfamily/Otubains (OTUB1 and OTUB2), the OTUD subfamily (OTUD1, OTUD2/YOD1, OTUD3, OTUD4, OTUD5/DUBA, OTUD6A, OTUD6B, ALG13, and HIN1L), the A20-like subfamily (A20, Cezanne, Cezanne2, TRABID, and VCPIP), and the OTULIN subfamily (OTULIN and FAM105A) (Figure 1A). The size of the catalytic domain distinguishes subfamilies—OTUD enzymes being the smallest (~150 amino acids [aa]), and the OTUB/OTULIN (220–270 aa) and A20-like OTUs (300–350 aa) containing larger catalytic folds. Most human OTUs contain additional domains, including UBDs (Figure 1B).

We cloned the 16 catalytic-triad-containing human OTU DUBs from plasmids, IMAGE clones, or human complementary DNA (cDNA) libraries and expressed and purified full-length (FL) and/or OTU domain-containing constructs in *E. coli* (Figures 1C and 1D). Most OTUs reacted quantitatively with Ub propargylamide (Ub-PA) (Ekkebus et al., 2013), indicating proper folding and a reactive catalytic Cys (Figure 1E, Figure S1A available online). OTUD5/DUBA required activation by phosphorylation in the OTU domain by recombinant CK2 to display reactivity (Huang et al., 2012). OTULIN did not react with Ub-PA because it requires activation by a proximal Ub for activity (Keusekotten et al., 2013). ALG13 did not react with Ub-PA, but it did react with haloalkyl probes, and A20 reacted very slowly and incompletely with all tested probes (Figures 1E, S1A, and S1B).

The C terminus of Ub is important for DUB reactivity (Drag et al., 2008). The Ub-like modifiers ISG15 and NEDD8 have identical or similar C-terminal sequences, and whereas OTUB1 is Ub specific (Edelmann et al., 2009), viral OTU domains (vOTU) can be cross-reactive for Ub and ISG15 (Frias-Staheli et al., 2007). We found that ISG15-based suicide probes that modified vOTU (Akutsu et al., 2011) did not react with human OTU DUBs (Figure S1C). In contrast, 13 of the 16 human OTU DUBs were modified by NEDD8-derived suicide probes to varying degrees (Figure S1D). However, comparing Ub- and NEDD8-based peptide substrates in fluorescence polarization assays (Geurink et al., 2012) (see below) showed that OTU DUBs only hydrolyzed the Ub-based, but not the NEDD8-based, substrates under identical conditions (Figure S1E), indicating that human OTU DUBs are Ub specific.

Linkage Specificity of OTU DUBs against Diubiquitin

Next, we analyzed the linkage specificity of human OTU DUBs against all eight types of diubiquitin (diUb) (Figure 2A). Time-course experiments were performed at constant substrate concentration. Enzymes were used at different concentrations in order to identify the lowest DUB concentration that resulted in significant cleavage of the preferred chain type(s), indicating linkage preference of the DUB.

The results of this analysis revealed a striking and unexpected linkage specificity of all human OTU DUBs (Figure 2A). Six DUBs (Cezanne, Cezanne2 – Lys11; OTUD4, OTUB1 – Lys48; OTUD1 – Lys63; OTULIN – Met1) cleaved only one diUb substrate (group I), four DUBs (OTUD3 – Lys6 and Lys11; A20, VCPIP – Lys11 and Lys48; phosphorylated OTUD5 – Lys48 and Lys63) cleaved two substrates (group II), and four DUBs (OTUD2,

Figure 1. Human OTU DUBs and Reactivity of Analyzed Constructs

(A) Phylogenetic tree of human OTU domain DUBs. HIN1L (*) is a pseudogene, and FAM105A (**) is lacking active site residues.

(B) Domain composition in human OTU DUBs (updated from Komander et al. [2009]).

(C) Constructs analyzed in this study. Full-length proteins not used in this study are shown in gray.

(D) Purified OTU proteins according to (C) resolved on a Coomassie-stained 4%–12% SDS-PAGE gradient gel. M, marker. Asterisks (*) indicate purified constructs.

(E) Reactivity of analyzed constructs against the suicide probe Ub propargylamide (Ub-PA, upper panel) and Cy5-labeled Ub-PA (lower panel). Asterisks (*) indicate the modified form of the OTU DUB.

See also Figure S1.

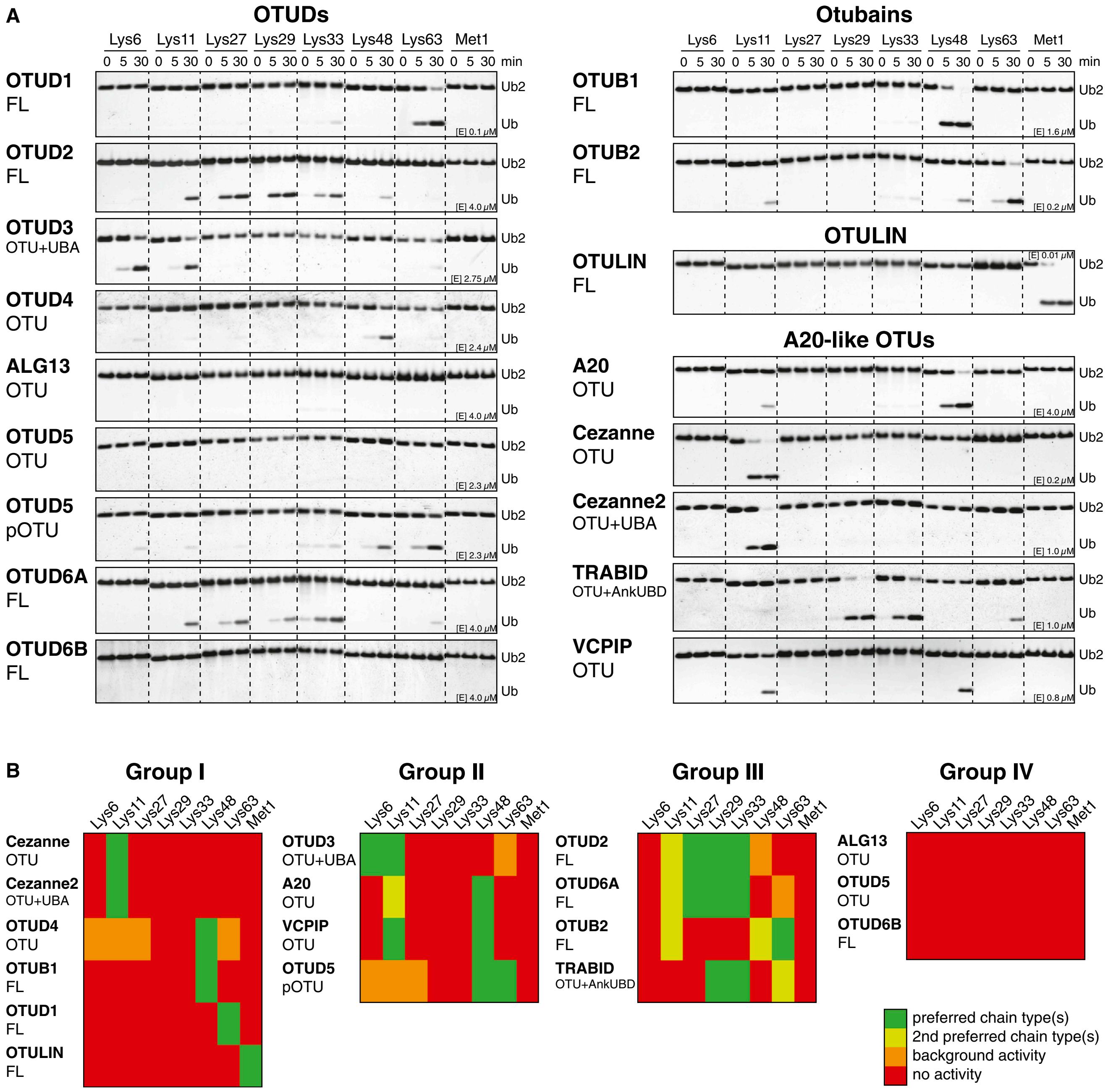

Figure 2. Linkage Specificity of Human OTU DUBs

(A) Purified OTU DUBs (constructs according to Figure 1C) were incubated with diUb of all linkage types for the indicated times and resolved on silver-stained SDS-PAGE gradient gels. Enzyme concentration is as indicated and differs for each DUB. See Figure S2 for additional experiments.

(B) OTU DUB linkage specificity against diUb substrates can be grouped to enzymes cleaving one linkage type (group I), two linkage types (group II), three or more linkage types (group III), or inactive enzymes (group IV).

OTUD6A, OTUB2, TRABID) cleaved three or more chains preferentially (group III) (Figures 2A and 2B). ALG13, unphosphorylated OTUD5, and OTUD6B were inactive in this assay (group IV) despite being modified by Ub suicide probes (Figures 1E, S1A, and S1B).

Increasing the concentration of DUB in the assay or using longer incubation times led to the hydrolysis of linkages other than the preferred linkage types (Figure S2). With the exception of OTULIN, no tested DUB hydrolyzed Met1 linkages even at a higher enzyme concentration or later time points (Keusekotten et al., 2013), suggesting that OTU DUBs are mostly isopeptidases. The OTU DUB cleavage profiles differed from USP domain DUBs that cleave all types of diUb with similar activity (Faesen et al., 2011).

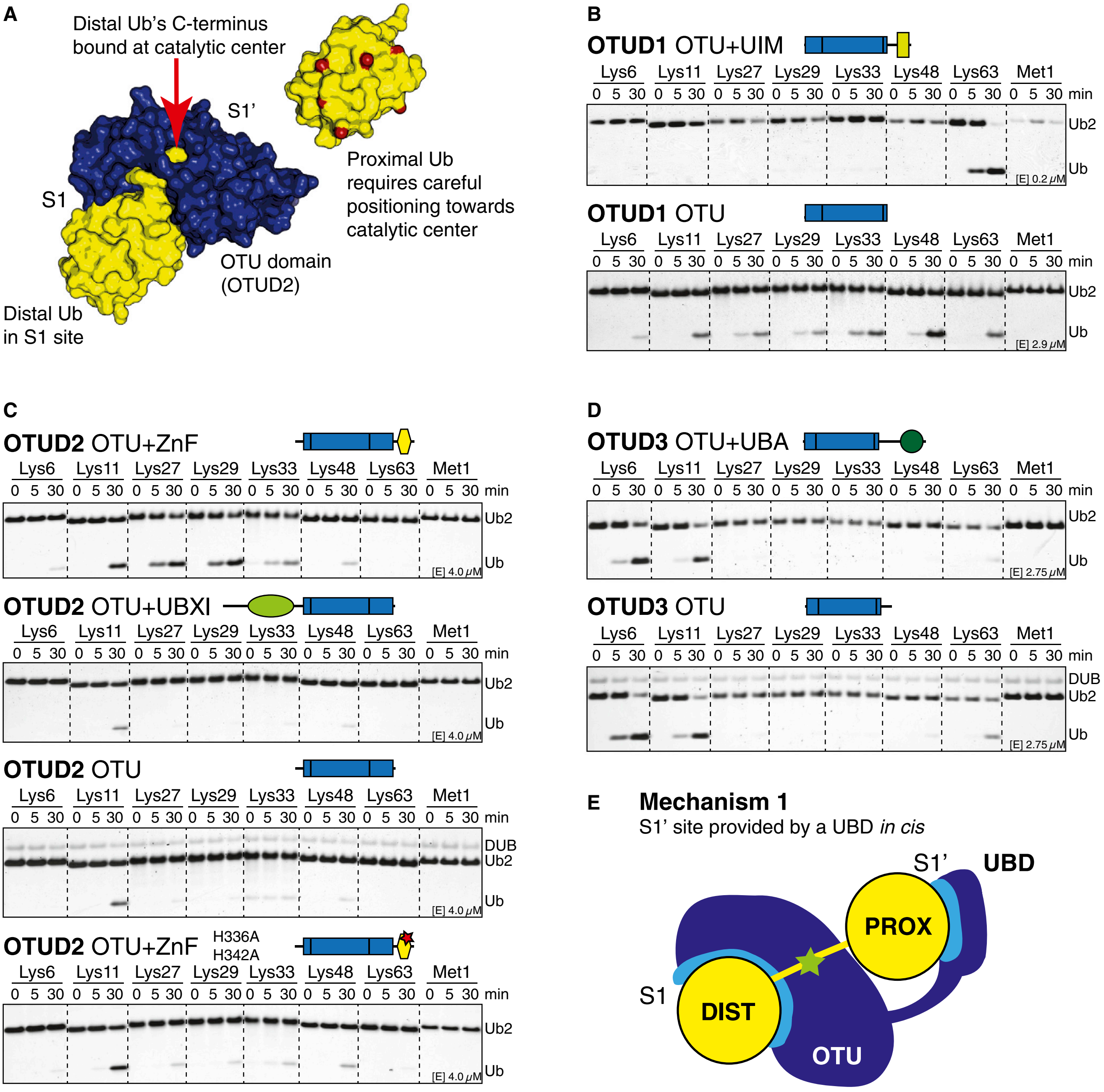

Figure 3. Roles for UBDs in OTU Specificity

(A) Surface representation of an OTU domain (blue) bound to a distal Ub molecule (yellow) with its C terminus reaching to the active site. The proximal Ub in the dimer needs to bind such that only the preferred linkage point(s) (indicated in red on Ub surface) are presented to the active site.

(B) DUB assays performed as in Figure 2A with OTUD1 aa 287–481 (OTU+UIM, top) and 287–437 (OTU, bottom). The construct lacking the UIM domain is nonspecific and less active (14.5× higher enzyme concentration used in gel below).

(C) Specificity analysis of different OTUD2 constructs. Top, OTUD2 lacking the UBX-like domain. Second from top, OTUD2 lacking the ZnF domain. Third from top, OTUD2 isolated OTU domain. Bottom, OTUD2 with a mutation in the ZnF domain. The ZnF affects the ability of OTUD2 to cleave Lys27-, Lys29-, and Lys33-linked diUb. See Figure S3 for additional experiments.

(D) Specificity assays of OTUD3 for constructs including the OTU and UBA domains (top) and the catalytic domain alone (bottom). The UBA domain has no influence on diUb hydrolysis.

(E) Mechanism 1, positioning and orientation of the proximal Ub is achieved by its binding to a UBD present in the OTU enzyme.

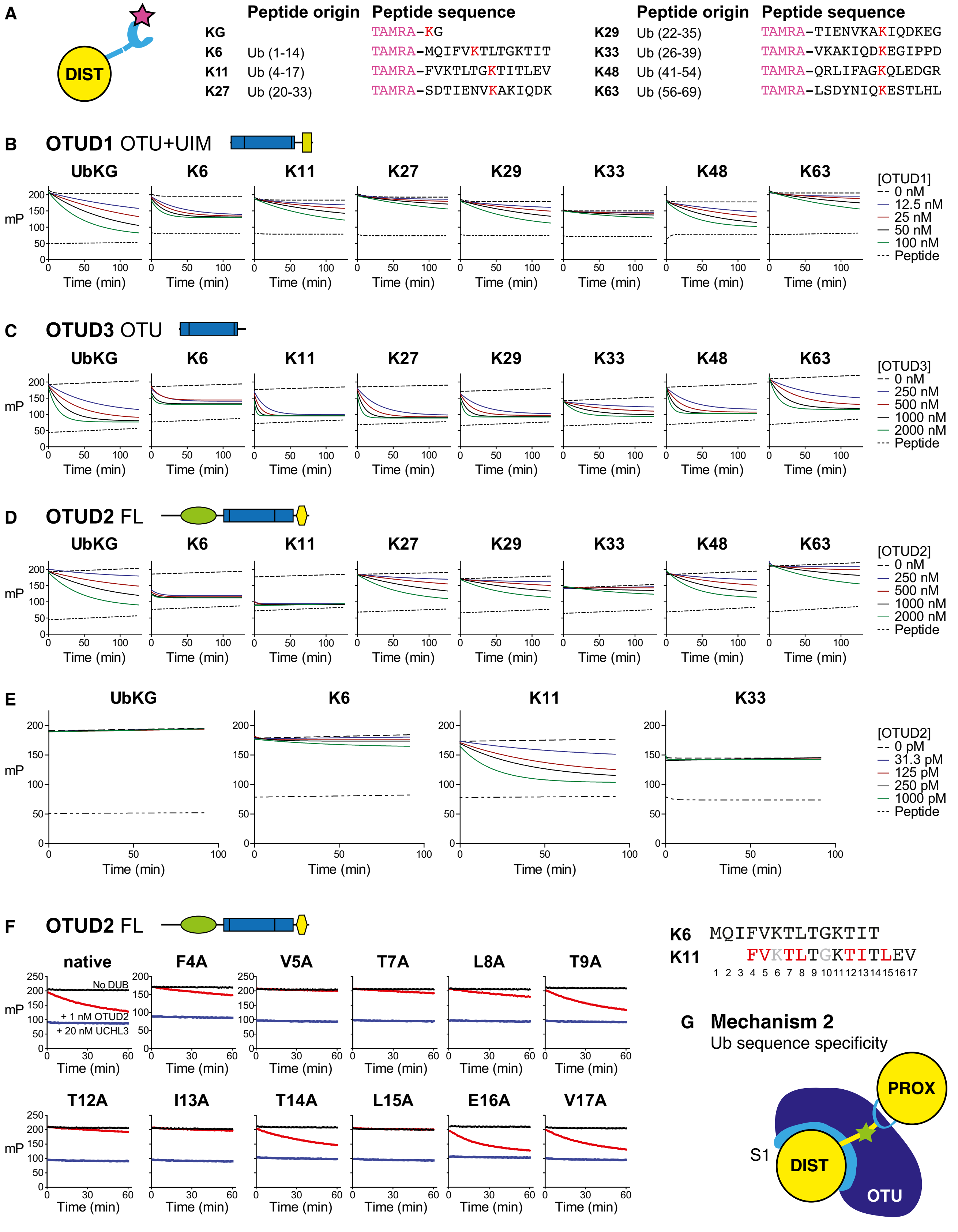

(legend on next page)

It was unclear whether OTU orthologs have conserved their linkage preference throughout evolution. *S. cerevisiae* encode only two OTU DUBs, yOtu1 and yOtu2. yOtu1 and *D. melanogaster* (dm) Otu1 are orthologs of human OTUD2 (38% and 53% identical in OTU domain, respectively), and OTUD2 and yOtu1 both bind cdc48/p97 and are involved in endoplasmic-reticulum-associated protein degradation (Ernst et al., 2009; Rumpf and Jentsch, 2006). OTUD2 and yOtu1 preferred the same atypical linkages (Figures 2A and S2D), whereas dmOtu1 also cleaved Lys6 linkages, indicating that the linkage profiles of OTU enzymes are not necessarily identical in different species (Figure S2E).

Altogether, this revealed that the OTU family had evolved enzymes that recognize and hydrolyze specific Ub chain types.

Mechanisms of Linkage Specificity

Distinct Ub linkage specificity in members from a single DUB family was unexpected and required a mechanistic explanation. During the hydrolysis of diUb, both Ub moieties interact with the DUB's catalytic domain (Figure 3A). The distal Ub moiety binds to the enzymatic S1 site and positions its C-terminal tail in the catalytic site. This distal Ub is identical in each diUb molecule and does not explain linkage specificity. In contrast, the proximal Ub moiety that binds to the enzymatic S1' site contributes the Lys to the isopeptide bond. Hence, mechanisms to position and orient the proximal Ub moiety are the key to understand linkage specificity in DUBs.

We selected three members of the OTUD family for additional investigation: the unstudied Lys63-specific OTUD1, the cdc48/p97 interactor OTUD2 that cleaves atypical linkages (Lys11, Lys27, Lys29, and Lys33), and OTUD3, another unstudied DUB with activity against Lys6- and Lys11-linked diUb (Figure 2A).

Roles for UBDs in Linkage Specificity

First, we tested whether UBDs in OTUDs contribute to positioning the proximal Ub toward the catalytic center. OTUD1 contains a C-terminal Ub-interacting motif (UIM, aa 457–476), OTUD2 contains an UBX-like domain (aa 46–128) and a C-terminal zinc finger (ZnF, aa 318–342), and OTUD3 contains a C-terminal Ub-associated domain (UBA, aa 230–270). We compared the activity and linkage specificity for truncated OTUD enzymes (Figure 3).

The removal of the OTUD1 UIM had dramatic effects on activity and linkage specificity. Full-length OTUD1 or a construct comprising OTU and UIM were highly active and Lys63 specific (Figures 2A and 3B). The removal of the UIM in the OTU-only construct rendered the protein less active (assay performed at a 14.5× higher enzyme concentration) and, importantly, nonspecific (Figure 3B). Hence, in OTUD1, the UIM greatly increased the specificity and efficiency of the enzyme toward Lys63 linkages. This is similar to TRABID, where an N-terminal ankyrin-repeat Ub-binding domain is required for Lys29 and Lys33 linkage specificity (Licchesi et al., 2012).

Full-length OTUD2 cleaved Lys11-, Lys27-, Lys29-, and Lys33-linked diUb (Figure 2A). Removal of the N-terminal UBX-like domain did not affect OTUD2 specificity, but deletion of the C-terminal ZnF domain or point mutations in zinc-binding residues significantly reduced activity toward Lys27-, Lys29-, and Lys33-linked diUb without affecting Lys11 activity (Figure 3C). The same was observed in dmOtu1 (Figure S3A). Hence, the ZnF domain in OTUD2 enabled a Lys11-specific catalytic core domain to cleave three additional linkage types. This suggested that the OTUD2 ZnF is a UBD; however, we were unable to detect an interaction with monoUb in nuclear magnetic resonance (NMR) chemical shift perturbation experiments (Figures S3B–S3E). UBDs do not always influence linkage specificity, at least for diUb substrates, as shown for OTUD3, where the removal of the UBA domain did not change its ability to cleave Lys6- and Lys11-linked diUb (Figure 3D).

Hence, additional domains can both restrict and broaden the linkage specificity profile of OTU DUBs and fulfill important roles in regulating OTU activity and linkage specificity (Figure 3E). Notably, 8 of the 16 human OTU DUBs contain UBDs (Figure 1B), suggesting that this could be a widely used mechanism. Moreover, UBDs in DUBs of other families (USPs and Josephins) could have similar roles.

Sequence Specificity in OTU Domain DUBs

Isolated catalytic OTU domains showed distinct linkage specificity against diUb substrates (Figures 3B–3D), and, next, we investigated whether the entire proximal Ub or only the sequence surrounding the ubiquitinated Lys was important for linkage specificity. For this, fluorescent ubiquitinated 14-mer peptides derived from Ub (Figure 4A) (Geurink et al., 2012), as well as a minimal fluorescent Lys-Gly (KG) peptide, were used in fluorescence anisotropy assays at fixed substrate and increasing OTU DUB concentrations (Figures 4 and S4).

In the majority of OTU DUBs tested, the peptide probes did not reflect the linkage specificity seen with diUb. OTUD1, OTUD3, OTUB1, and Cezanne2 hydrolyzed most or all peptide

Figure 4. Linkage Specificity Determinants in the Proximal Ub

(A) Schematic representation (left) and sequence of generated fluorescent ubiquitinated Ub peptides. The red K indicates the ubiquitination site in the peptide. TAMRA refers to the fluorescent group appended to the N terminus of the peptide.

(B–E) OTUD1 (B), OTUD3 (C), and OTUD2 (D and E) used at the different concentrations (indicated to the right) cleaved the indicated peptides over time. OTUD1 (B) and OTUD3 (C) (as well as OTUB1 and Cezanne2, see Figure S4) hydrolyzed most of or all the peptides similarly, indicating a lack of sequence preference and a requirement for other regions in the proximal Ub to recover specificity. OTUD2 hydrolyzed all peptides if used at high enzyme concentrations (D) yet showed the highest activity against the K6 and K11 peptide that were already hydrolyzed at the start of the measurement. Dilution of OTUD2 to picomolar concentrations (E) revealed that the enzyme was sequence specific for a ubiquitinated peptide based on the Ub Lys11 context.

(F) Alanine scanning mutagenesis of the K11 peptide and assay with OTUD2 at 1 nM concentration as performed in (E). The y axis scale is the same in all graphs except F4A. Residues affecting OTUD2-mediated hydrolysis are indicated in red in the sequence alignment (right). Leu15, not present in the K6 peptide, explains the difference in sequence specificity between these similar peptides. Mutation of Lys6 to Ala resulted in an insoluble peptide, and Gly10 was not mutated. See also Figure S4.

(G) Mechanism 2, OTUD2 is able to read the sequence context of the ubiquitination site, bind, and cleave in a sequence-specific fashion.

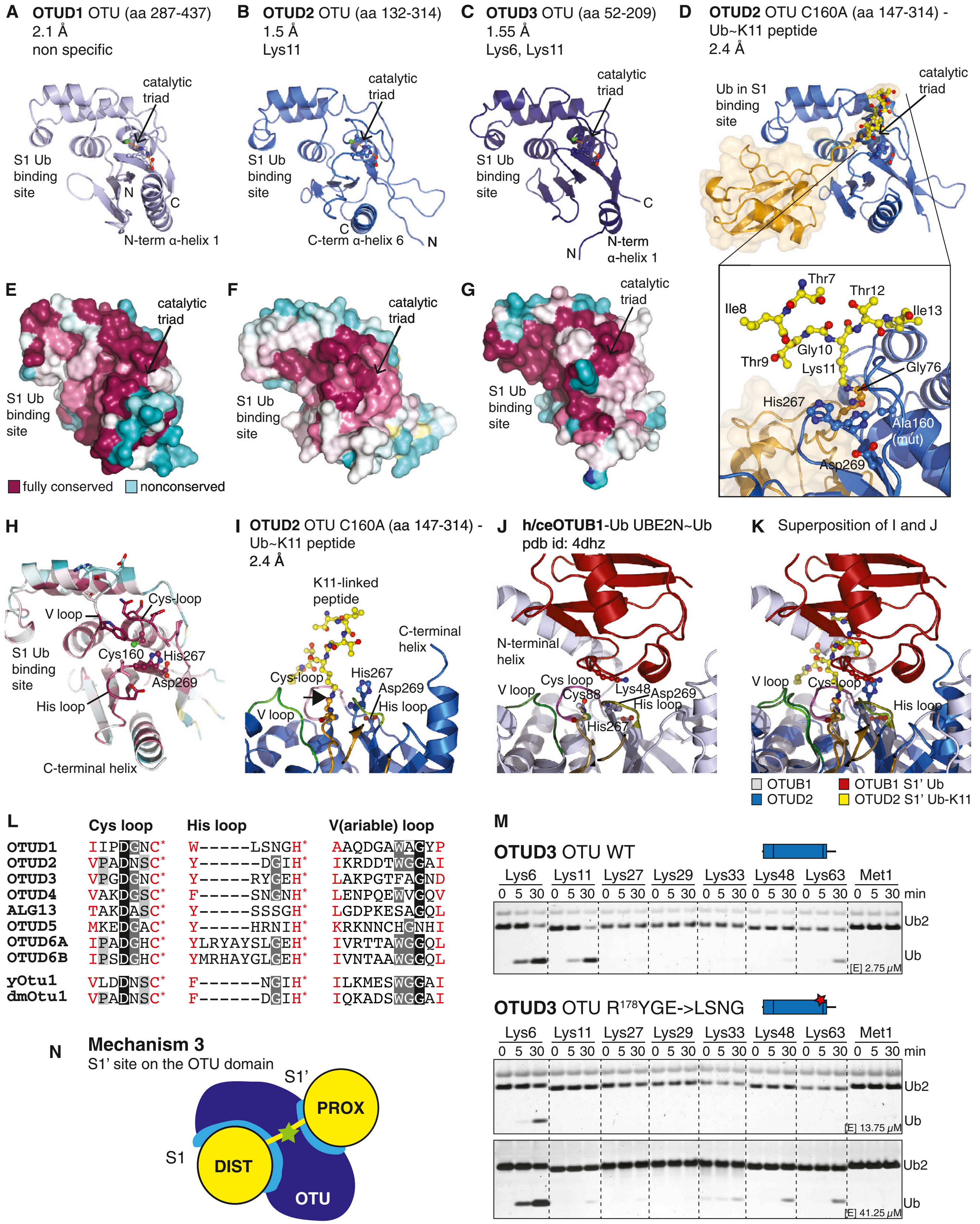

(legend on next page)

substrates (Figures 4B, 4C, S4A, and S4B), albeit with reduced activity for some combinations (e.g., OTUD1 against K33 peptide, Figure 4B). This suggested that the recognition of the entire proximal Ub fold is required for the linkage specificity of these DUBs, which was consistent with the involvement of, for example, UBDs (Figure 3).

In contrast, and to our surprise, OTUD2 displayed a marked specificity for the peptide that was derived from the Lys11 sequence of Ub (K11 peptide, Figures 4D and 4E). OTUD2 hydrolyzed all peptide substrates at a high enzyme concentration but had already completely hydrolyzed the K11 peptide at the start of the measurement (Figure 4D). Dilution of OTUD2 to picomolar concentrations recovered complete specificity of the DUB against the K11 peptide, and even the similar K6 peptide was not hydrolyzed significantly at low enzyme concentrations (Figure 4E). To further understand this, we mutated each amino acid of the ubiquitinated K11 peptide to Ala (Figure S4C). The K6A peptide was insoluble, and Gly10 was not mutated. Experiments performed at an OTUD2 concentration that cleaved the K11 peptide revealed that Ala substitutions of Phe4, Val5, Thr7, Leu8, Thr12, Ile13, and Leu15 significantly reduced the hydrolysis activity of the peptide (Figures 4F and S4D). Several of these residues are solvent exposed in Ub, suggesting that OTUD2 binds to these residues of the proximal Ub. However, Ile13 and Leu15 are not exposed in folded Ub and, hence, are unlikely to play a role in diUb recognition.

Nonetheless, this revealed another mechanism of OTU DUB linkage specificity whereby OTUD2 selected the sequence context of a ubiquitinated substrate, in this case recognizing the Ub sequence surrounding Lys11 (Figure 4G).

Structural Studies on OTUD Family DUBs

To understand the specificity of OTUD domains at the molecular level, we determined high-resolution crystal structures of OTUD1 (aa 287–437, 2.1 Å, Figures 5A and S5A), OTUD2 (aa 132–314, 1.5 Å, Figures 5B and S5B), and OTUD3 (aa 52–209, 1.55 Å, Figures 5C and S5C) (Table S1). The catalytic domains are structurally similar to each other and to OTUD5 (Huang et al., 2012) and *S. cerevisiae* Otu1 (yOtu1) (Messick et al., 2008), root-mean-square deviations (rmsds) being from 0.6–1.0 Å (Figure S5D). Catalytic triads are in competent conformations, as observed for pOTUD5 in complex with a Ub suicide probe (Huang et al., 2012) (Figures S5D and S5E).

Furthermore, we determined the structure of OTUD2 bound to the ubiquitinated K11 peptide (Figure 5D), representing the first structure of an OTU with an isopeptide bond spanning the active site. Clear electron density for the isopeptide bond (Figure S5F) and for four residues upstream and two residues downstream of the ubiquitinated Lys revealed how the scissile bond reaches across the active site. Unfortunately, the close packing of a symmetry-related molecule (Figure S5G) most likely affects the position of the peptide, and residues that affect K11 peptide hydrolysis (Phe4, Val5, and Leu15) (Figures 4F and S4D) are disordered in the structure. The peptide does not form significant contacts with the protein, which would have been expected from the peptide assay, suggesting that crystal lattice formation affects peptide binding.

The Ub in the OTUD2 K11 peptide structure is located at a similar position in the S1 site of the enzyme in comparison to structures of OTUDs with Ub-based suicide inhibitors (Huang et al., 2012; Messick et al., 2008) (Figures 5D and S5E). OTUD5, but not yOtu1, requires activation by phosphorylation in the OTU domain, which leads to the formation of the S1 Ub-binding site (Huang et al., 2012) (Figure S5E). In OTUD1, OTUD2, and OTUD3, the corresponding secondary structure elements are present with or without Ub bound (Figure S5D), and there are no large-scale conformational changes in OTUD2 upon Ub binding (Figures 5B and 5D).

Conserved and Distinct OTU Domain S1' Ub-Binding Sites

The K11 peptide structure revealed how the isopeptide bond is bound by OTU domains and how the proximal Ub is contacted to form an S1' substrate-binding site on OTUD DUBs. The Lys side chain approaches the catalytic center across the loop preceding the catalytic Cys, termed the Cys loop (Figures 5H, 5I, and S5H). The neighboring His loop connects the catalytic His with a conserved upstream aromatic residue that forms interactions with the C terminus of the distal Ub. A third loop, the variable loop (V loop), located opposite to the His loop

Figure 5. Structural Studies on OTUD1, OTUD2, and OTUD3 Reveal a Conserved S1' Site

(A–C) Crystal structures of the OTU domains of OTUD1 (A), OTUD2 (B), and OTUD3 (C). A cartoon representation in identical orientation is shown. The S1 Ub-binding site, N and C termini, and N- or C-terminal α helix are labeled.

(D) Structure of inactive OTUD2 catalytic domain (C160A) in complex with the ubiquitinated K11 peptide (orange, see Figure 4) bound across the active site of the enzyme (boxed) shown as in (B). The inset shows a stick model of the ubiquitinated peptide.

(E–G) Surface residues of OTUD1 (E), OTUD2 (F), and OTUD3 (G) are colored according to conservation of the protein throughout evolution (on the basis of the alignments in Data S1).

(H) Top view of the putative S1' site in OTUD2. The peptide structure in (D) reveals how the isopeptide is bound across the active site of an OTU DUB. Cys, His, and V loops as well as the C-terminal helix are indicated.

(I) Putative S1' site in the structure of OTUD2 bound to the ubiquitinated K11 peptide. An arrow indicates the scissile bond.

(J) The same view as in (I) for the OTUB1 structure with two Ub moieties bound in S1 and S1' sites (Wiener et al., 2012). The proximal Ub contacts the Cys and His loops and also a dedicated S1' binding site in a protruding N-terminal helix unique to OTUB1.

(K) Superposition of (I) and (J) showing the compatibility of S1' binding sites.

(L) Sequence of Cys, His, and V loops in the human OTUD enzymes, yOtu1, and dmOtu1. Residues in red are "anchor" points of conserved structural residues. An asterisk (*) indicates catalytic Cys or His.

(M) A His loop mutation in OTUD3, R^{178}YGE to LSNG, creates a less active OTUD3 variant in which Lys11-diUb activity is more strongly affected than Lys6-diUb activity (see also Figure S5K). Note the differences in enzyme concentration used in the assays.

(N) Mechanism 3, a conserved S1' Ub-binding site on OTU DUBs positions the proximal Ub toward the catalytic center.

See also Figure S5.

may also contact the proximal Ub. Along with these loops, the N-terminal helix in the OTUD1 and OTUD3 catalytic domain and the structurally equivalent C-terminal helix of the OTUD2 catalytic domain form the putative S1' site that binds the proximal Ub (Figures 5H, 5I, and S5H). In recent complex structures of OTUB1 with Ub bound in the S1' site of the DUB (Juang et al., 2012; Wiener et al., 2012) and of OTULIN bound to Met1-linked diUb (Keusekotten et al., 2013), additional N-terminal helices form extensive S1' sites (Figures 5J, 5K, S5I, and S5J). These are not present in minimal OTUD domains (Figures 5I and S5H).

When the sequence conservation of OTUD orthologs from species annotated in the Ensembl project (www.ensembl.org; Data S1) is mapped onto the surface of OTUD1, OTUD2, and OTUD3, the putative S1' site comprising Cys and His loops emerged as regions of highest surface conservation greater than the S1 Ub-binding site (Figures 5E–5G). Importantly, the amino acid sequence in the loops varies significantly between OTUD family members, in particular in the His and V loops (Figure 5L), indicating changes that may account for the observed differences in linkage specificity.

We wondered whether mutations in the His and Cys loops would change the cleavage profile of OTUD DUBs. Substitution of the His loop of OTUD3 by the corresponding sequence in OTUD1 (mutating R^{178}YGE to LSNG) rendered the protein significantly less active in comparison to the wild-type (WT) enzyme and affected its ability to target Lys11-linked, but not Lys6-linked, diUb, even at very high concentrations (Figures 5M and S5K). Hence, we engineered an OTU domain with a unique specificity profile against diUb.

Altogether, the structural and mutagenesis data revealed distinct S1' Ub-binding sites on OTUD family enzymes that contribute to their ability to target selected Ub linkages (Figure 5N). However, complex structures with diUb bound across the active site are required to fully understand OTUD specificity and to rationally design enzymes with new properties.

An S2 Site in OTUD2 Enables Specificity for Longer Lys11-Linked Chains

Our attempts to generate substrate-bound OTUD structures revealed an additional mechanism of specificity for OTUD2. In a structure of inactive OTUD2 C160A in complex with Lys11-linked diUb, the diUb molecule did not bind across the active site but occupied S1 and a previously unidentified S2 site on OTUD2 (Figure 6A). The S2 site is formed by two exposed hydrophobic residues (Ile292 and Val295) on the C-terminal OTUD2 α helix that bind the hydrophobic Ile44 patch of Ub (Figure 6B). The orientation of Ub bound to the S2 site most likely allows preferential binding of Lys11-polyUb, given that the S2 Ub points with its C terminus toward Lys11 of the S1 Ub (Figure 6A). Interestingly, in the structure of OTUD2 C160A bound to the ubiquitinated K11 peptide (Figure 5D), a second Ub in the asymmetric unit occupied the S2 site in an identical manner (Figure 6C). The S2 site in OTUD2 is conserved in higher eukaryotes but not in yOtu1 and dmOtu1 (Figure 6B and Data S1).

We tested whether the S2 site was functionally relevant in isolated catalytic domains of OTUD2 variants and mutated Ile292 and Val295 to Gln (referred to as OTUD2 MutS2), which did not affect reactivity or diUb specificity (Figures S6A and S6B). Next, we compared the activity of the OTU domains of OTUD2, OTUD2 MutS2, and dmOtu1 toward Lys11-linked chains. All proteins hydrolyzed Lys11-diUb similarly, but Lys11-linked tri- and tetra-Ub were more rapidly cleaved to di- and mono-Ub by WT OTUD2, whereas OTUD2 MutS2 or dmOtu1 did not show enhanced activity for longer Lys11-linked chains (Figures 6D and S6C). The accumulation of Lys11-linked diUb indicated that this product might be stabilized by binding the S1 and S2 sites on OTUD2, as was observed in the complex structures, although OTUD2 MutS2 did not show enhanced diUb cleavage. The S2 site specifically enhanced the cleavage of Lys11-linked polyUb, given that Lys6-, Lys48-, or Lys63-linked triUb were less well hydrolyzed by WT OTUD2 in comparison to MutS2 or were not hydrolyzed at all (Figures 6E and S6D). OTUD1 does not provide a structurally equivalent hydrophobic S2 site on its α1 helix and is not enhanced in cleaving longer chains (Figure S6E).

Hence the presence of an S2 site on the OTUD2 catalytic domain allows it to specifically target longer Lys11-linked chains, revealing an additional mechanism of OTU specificity (Figure 6F).

Linkage-Specific OTU DUBs Enable the Characterization of polyUb Chains

Biochemical tools that allow the identification of the Ub chain type on a substrate are limited. Mass spectrometry, linkage-specific antibodies, Ub chain sensors, and Ub mutants have been used to determine Ub chain type and topology, but all these methods have limitations (Kulathu and Komander, 2012; Williamson et al., 2013).

We tested whether linkage-specific OTU DUBs could be used in analogy to DNA restriction enzymes to hydrolyze specific linkages in complex samples to reveal the linkage type(s) present in a ubiquitinated substrate. In combination, OTU DUBs can be used to examine most linkage types (Figures 2 and 7A).

Using linkage-specific assembly systems, we generated Lys63-, Lys48-, Lys11- and Met1-polyubiquitinated model substrates in vitro, (see Experimental Procedures), which were treated with a panel of DUBs (Figures 7A–7G and S6F–S6H). Under these conditions, the nonspecific enzyme USP21 (Ye et al., 2011) hydrolyzed most or all ubiquitin linkages, whereas the nonspecific vOTU DUB (Akutsu et al., 2011) efficiently removed all isopeptide-linked polyUb.

Linkage-specific OTU DUBs were used at a low concentration in order to maximize DUB specificity, and they were also used at a 3×–10× higher concentration in order to drive preferred reactions to completion (Figure 7B). DUB-treated samples were resolved on SDS-PAGE gradient gels and analyzed by silver staining and/or western blotting. Three parameters indicated that DUBs affected the substrate: (1) the reduction of high-molecular-weight (HMW) polyUb, (2) the emergence of monoUb, and (3) the appearance of free chains released from HMW species.

The OTU DUBs cleaved polyUb substrates according to their specificity profiles. OTUD1 reduced Lys63-polyUb to monoUb (Figures 7C, 7D, S6F, and S6G), and OTUB1 generated monoUb from E6AP-assembled Lys48-polyUb (Figures 7E and S6H). OTUD3, Cezanne, and OTUD2 hydrolyzed UBE2S-assembled

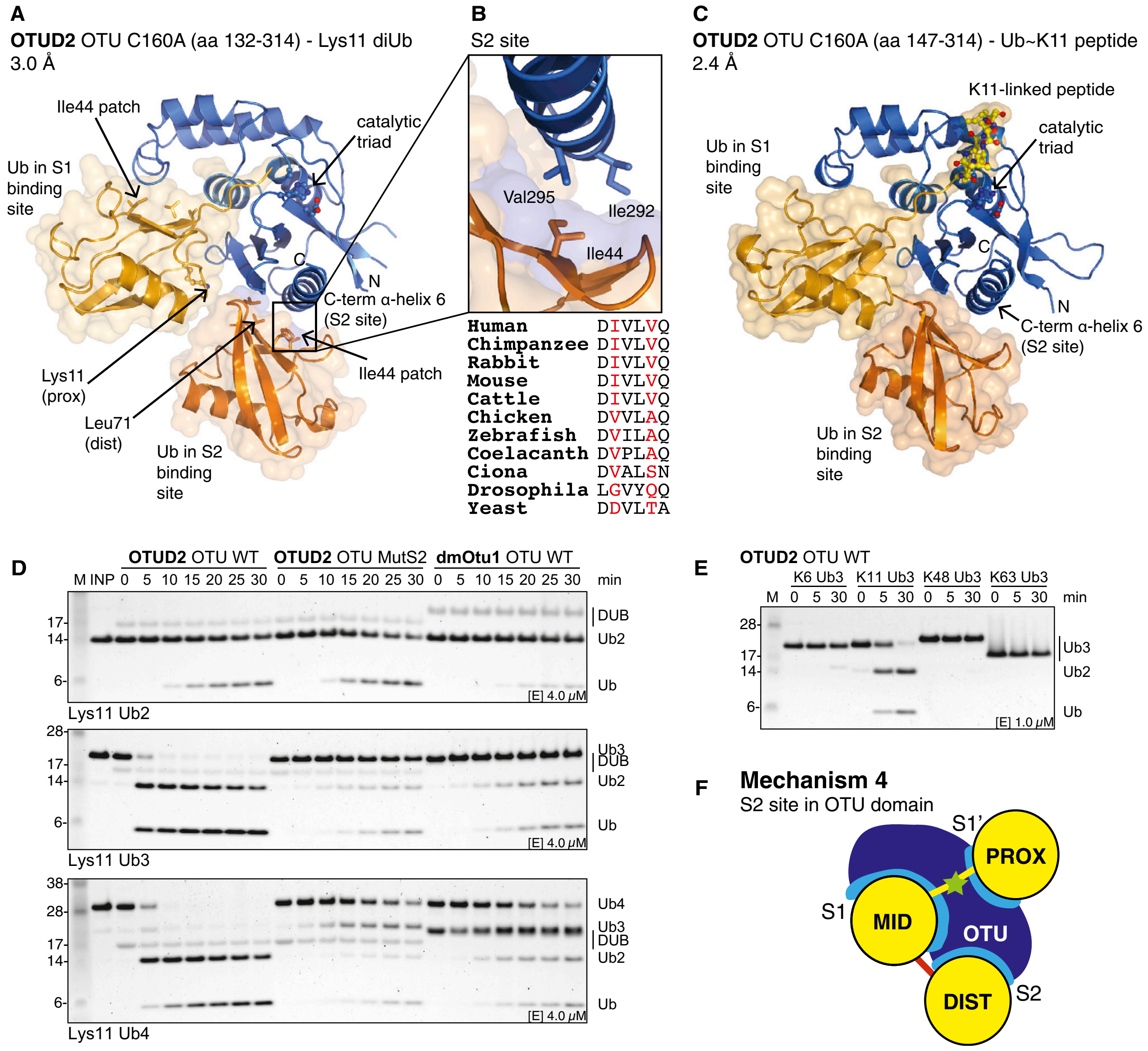

Figure 6. Complex Structures of OTUD2 Reveal an S2 Ub-Binding Site

(A) Structure of the inactive OTUD2 OTU domain (C160A) bound to Lys11-linked diUb in the S1 and S2 site of the enzyme. The orientation of the Ub molecules is compatible with Lys11 linkage, although the linker sequence is not resolved in the electron density maps (indicated by arrows).

(B) Close-up image of the hydrophobic S2 site on the α6 helix formed by Ile292 and Val295, which interact with the Ile44 patch of Ub. An alignment shows conservation of this sequence in different species (see also Data S1).

(C) Structure of inactive OTUD2 in complex with the ubiquitinated K11 peptide as in Figure 5D. A second Ub for which the peptide is disordered is bound in the S2 site (see Figure S5G).

(D) DUB assays with Lys11-linked Ub chains. Assays comparing isolated catalytic domains of WT OTUD2 (aa 147–314) and S2 site mutant (MutS2, aa 147–314, I292Q, V295Q) as well as dmOtu1 (aa 143–313) toward Lys11-diUb (top), Lys11-triUb (middle), and Lys11-tetraUb (bottom). Human OTUD2 hydrolyzed tri- and tetra-Ub immediately, and this depended on the S2 site of the enzyme.

(E) Cleavage of differently linked triUb chains. In comparison to (D) and Figure 2A, a 4-fold lower enzyme concentration was used.

(F) Mechanism 4, an S2 Ub-binding site on OTU DUBs allows the DUB to target and specifically hydrolyze longer Ub chains.

See also Figures S6A–S6E.

Lys11-linked chains, and diUb accumulated in OTUD2-treated samples (Figure 7F). Only OTULIN hydrolyzed HOIP-assembled Met1-linked chains (Figure 7G).

Interestingly, in some cases, OTUD DUBs released intact polyUb chains from substrates (Figures 7C–7E and S6F–S6H). This could be due to the presence of chain types other than

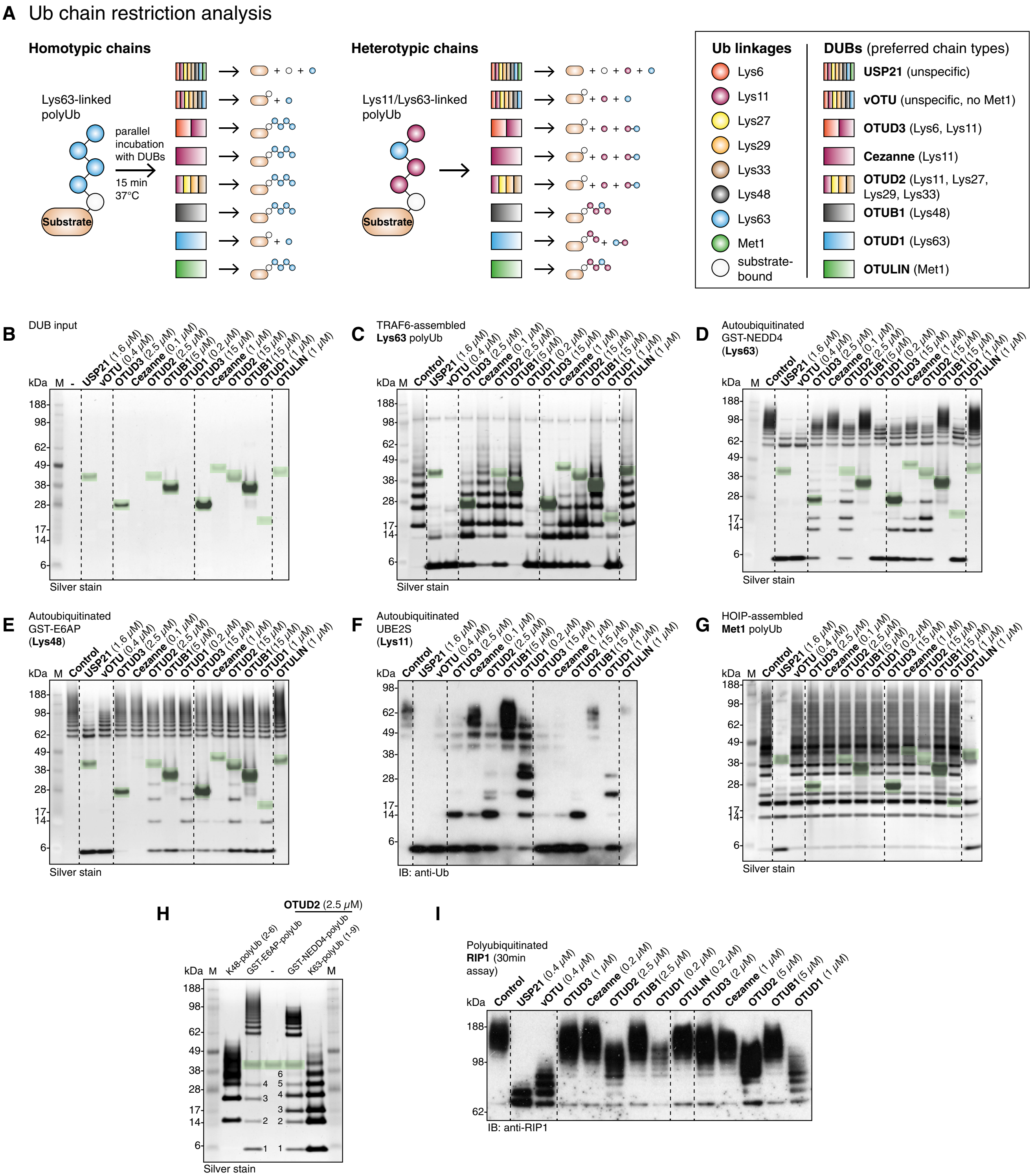

Figure 7. Exploiting OTU DUBs in Ub Chain Restriction Analysis

(A) Schematic of the principle of Ub chain restriction analysis.

(B–I) Ub chain restriction analysis against the indicated substrates. SDS-PAGE gradient gels were silver-stained (B–E, G, and H) or western blotted with anti-Ub (F) or anti-RIP1 (I). M, marker; Control, ubiquitinated protein without DUB treatment. Enzyme bands are highlighted in silver-stained gels (green boxes).

(B) Enzyme input reference gel.

(C) GST-TRAF6, UBE2N, and UEV1A generated free and attached Lys63-linked polyUb. See Figure S6F for an anti-Ub western blot of this gel.

(legend continued on next page)

the preferred chain types in assembly reactions, cleavage of the isopeptide linkage between substrate and Ub chain, or the hydrolysis of branched Ub polymers. Importantly, released intact polyUb chains could still be used to identify chain types, given that differently linked polyUb chains have distinct electrophoretic mobility. OTUD2 released chains from GST-tagged NEDD4 and E6AP, which showed identical electrophoretic mobility to free Lys48- or Lys63-linked polymers, respectively (Figure 7H). A double band for triUb observed in a OTUD2-treated UBE2S sample indicated small amounts of Lys63 linkages in the reaction, as reported previously (Bremm et al., 2010).

In the case of GST-E6AP, DUB treatment was inefficient, and HMW species remained, even at high concentration of DUBs (Figure 7E). This is consistent with recent data showing that longer Lys48 chains may be more resistant to DUB hydrolysis (Schaefer and Morgan, 2011; Ye et al., 2012).

Altogether, our data showed that OTU DUBs maintained their specificity when tested against polyubiquitinated substrates. To test their action against endogenously ubiquitinated substrates, we purified the TNF receptor signaling complex (TNF-RSC) using FLAG-tagged TNFα. The TNF-RSC contains many ubiquitinated proteins, including RIP1, which can be detected by western blotting with an antibody against RIP1 (Figure 7I) and was previously shown to be modified with at least four different Ub chain types (Gerlach et al., 2011). When treated with the DUB panel, OTUD1 substantially reduced HMW forms of RIP1, suggesting the prevalence of Lys63 linkages on RIP1. OTUD2 was also able to reduce the polyUb RIP1 signal, but, in this experiment, it cannot be assessed whether OTUD2 also released polyUb chains. In comparison, Cezanne, OTUB1, and OTULIN treatment did not lead to a strong reduction of the polyUb signal (Figure 7I), suggesting that Lys11-, Lys48-, and Met1-linked chains only account for a small fraction of the total linkages in RIP1.

Altogether, these experiments showed that OTU DUBs can be used to interrogate the type and relative abundance of Ub chains on substrates. We believe that Ub chain restriction analysis will be a useful tool in Ub chain research.

DISCUSSION

OTUs: A Remarkable DUB Family

Deubiquitinases are the subject of intense research, and many are intimately linked to human disease. Here, we characterized the second largest human family of DUB enzymes biochemically and structurally to discover that individual OTU DUBs have evolved distinct Ub linkage specificities. This finding is in contrast to USP DUBs, which cleave most Ub chain types indiscriminately (Faesen et al., 2011) and to JAMM family enzymes, many of which are Lys63 specific (Cooper et al., 2009). This insight immediately suggests that OTU DUBs may be less specific to the ubiquitinated protein per se and that their role is to regulate the abundance of selected Ub chain types that may arise under certain physiological conditions.

Four Mechanisms of Ub Linkage Specificity

We identify four distinct mechanisms of how OTU DUBs achieve linkage specificity. Of these mechanisms, two rely on proper positioning of the proximal Ub, which is achieved by either additional UBDs or an S1’ Ub-binding site on the OTU domain itself. Future structural studies of DUB polyUb complexes may allow DUB specificity engineering to generate enzymes with improved specificity, which would be beneficial for Ub chain restriction analysis and deeper understanding Ub chain biology.

Furthermore, we found that most OTU DUBs hydrolyze ubiquitinated Ub-derived peptides nonspecifically, indicating that an intact proximal Ub is required for their linkage specificities, which is consistent with aforementioned mechanisms. Interestingly, OTUD2 was highly selective for a ubiquitinated peptide derived from the Lys11 context of Ub, and an Ala scan revealed the residues involved in this specificity. Some of these residues (Ile13 and Leu15) are not exposed in Ub and do not explain the observed chain specificity but indicate that hydrophobic patches are most likely involved in proximal Ub recognition. The identification of a seemingly sequence-specific DUB fuels an ongoing debate on sequence specificity in protein ubiquitination. Global proteomic studies indicate a lack of sequence preference in protein ubiquitination sites (Kim et al., 2011; Wagner et al., 2011), and current models suggest that E3 ligases target a “ubiquitination zone” on substrates to modify accessible Lys residues within reach of the E3 ligase. However, the anaphase promoting complex (APC/C) preferentially ubiquitinates an initiation motif in its substrates (Williamson et al., 2011), suggesting that ubiquitination may, in some cases, be sequence specific.

Our structural studies of Ub and diUb complexes for OTUD2 unexpectedly revealed another mechanism that targets OTUD2 to longer Ub chains. Both complex structures uncovered an S2 site on the OTU domain itself, and our functional studies indicate that this site provides a mechanism for enhancing activity, and therefore specificity, toward longer K11-linked Ub chains. It is possible that the UBDs in other OTU DUBs (and in other DUB families), may serve similar roles in targeting the enzymes to polyUb-modified substrates. These four mechanisms, along with the mechanism of substrate-assisted catalysis in OTULIN (Keusekotten et al., 2013), provide a basis for understanding linkage specificity in DUBs.

Physiological Questions Arising

Many members of the OTU family have remained relatively unstudied. The best-studied OTU enzymes are the Ub-chain-editing enzyme A20, an important negative regulator of NF-κB signaling (Hymowitz and Wertz, 2010), and OTUB1, a protein

(D) Lys63-autoubiquitinated GST-tagged NEDD4 HECT domain with UBE2L3. See also Figure S6G.

(E) Lys48-autoubiquitinated GST-E6AP with UBE2L3. See also Figure S6H.

(F) Lys11-autoubiquitinated UBE2S containing contaminating Lys63 linkages (Bremm et al., 2010).

(G) Met1-linked polyUb generated by a minimal HOIP construct with UBE2L3.

(H) OTUD2 released polyUb chains from GST-E6AP and GST-NEDD4 compared to free Lys48- and Lys63-polyUb.

(I) Ub chain restriction analysis of polyubiquitinated RIP1 generated by FLAG-TNFα mediated purification of TNF-RSC from human embryonic kidney 293T cells.

with roles in the DNA damage response (Nakada et al., 2010). Both enzymes prefer Lys48-linked polyUb, but it is not clear whether this linkage specificity is relevant for their function. Current models require A20 to hydrolyze Lys63 linkages (Hymowitz and Wertz, 2010), whereas OTUB1 was shown to have noncatalytic functions by acting as a cellular buffer for charged E2 enzymes (Nakada et al., 2010). Particularly for A20, the low activity for the OTU domain suggests that, for example, posttranslational modifications or one of the Ub-binding A20-interacting proteins (Hymowitz and Wertz, 2010) could modulate its activity and/or specificity.

An interesting observation is the specificity observed for OTUD2 and VCPIP. Both enzymes efficiently cleave Lys11-linked chains and interact with p97 (Ernst et al., 2009; Wang et al., 2004), suggesting that p97 may act on substrates containing atypical linkage types, although the role of DUBs in p97 function is not well understood (Tsai and Weissman, 2011). The similar specificity of OTUD2 and OTUD6A, another unstudied OTU DUB, may suggest functional similarities. An additional enzyme worth studying is OTUD3, given that it is, so far, the only DUB that cleaves Lys6-linked diUb with some degree of specificity. Lys6-linked polyUb is an enigmatic chain type for which cellular roles are currently unclear (Kulathu and Komander, 2012). Understanding the relevant interactions of OTU DUBs (Sowa et al., 2009) may indicate physiological functions for unstudied atypical Ub chain types.

OTUs as Tools in Ub Chain Research

We are excited by the prospects of Ub chain restriction analysis in which linkage-specific OTU DUBs are used in vitro to reveal the identity of the Ub chain type(s) on proteins, and we have recently reported that they are useful reagents to interrogate chain architecture in heterotypic chains (Hospenthal et al., 2013). However, there are several caveats. The amount of polyubiquitinated substrate is often unclear, especially in western blotting applications. Also, the length, complexity, and number of Ub chains on in vitro generated polyubiquitinated proteins are often unknown. Therefore, each application of restriction analysis requires careful titration of each DUB to prevent off-target reactions. This is exaggerated when DUB activity depends on chain length (as seen for OTUD2). Furthermore, it is currently unclear whether OTU DUBs can hydrolyze the first Ub linkage (between substrate and proximal Ub) and how OTU DUBs deal with branched polyUb (in which one Ub is modified at two or more Lys residues, generating a forked structure). OTUB1 and OTUD3 hydrolyze heterotypic (mixed and branched) and homotypic chains equally well (Hospenthal et al., 2013; Nakasone et al., 2013).

Although some OTU DUBs seem remarkably specific (OTUB1 does not hydrolyze Lys6 linkages, even at high concentration in overnight reactions) (Hospenthal et al., 2013), the small OTUD family enzymes will cleave any linkage type when used at high concentrations or over long time courses. With a deeper understanding of OTU DUB mechanisms, specificity, and additional structural insights, efforts to "design" specificity in OTU DUBs may generate enzymes with improved specificity and activity. The use of Ub chain restriction analysis is not limited to OTU DUBs—other DUB families, in particular the Lys63-specific JAMM enzymes, could be excellent additional tools for these purposes.

In addition to Ub chain restriction analysis, there are several other ways linkage-specific OTU DUBs could be exploited; e.g., in mass-spectrometric applications to reveal proteins in lysates harboring particular chains types or when inactivated DUBs are used as linkage-specific UBDs to enrich certain linkage types. Clearly, OTU family DUBs will continue to be valuable tools in understanding the complex biology of protein ubiquitination events.

EXPERIMENTAL PROCEDURES

Cloning, Expression, and Purification of OTU DUBs

cDNAs for OTU DUBs were obtained from the IMAGE consortium by amplification from human cDNA libraries or as a gift from kind colleagues. Constructs according to Figure 1C were expressed in *E. coli* from pOPIN-K vectors and purified by affinity chromatography, anion exchange, and gel filtration.

Modification of OTU DUBs by Suicide Probes

Ub-PA was generated as described in Ekkebus et al. (2013) and Ub-, NEDD8-, and ISG15-derived haloalkyl probes were generated according to Akutsu et al. (2011) and Borodovsky et al. (2002). DUB reactivity assays were performed at room temperature for 1 hr (Ub-PA), 3 hr (haloalkyl probes), or as indicated.

In Vitro DUB Assays

Qualitative in vitro DUB linkage specificity assays were performed as in Licchesi et al. (2012).

Crystallization and Structure Determination

Crystallization screening was performed in a sitting drop setup with commercial screens. Structures were determined by molecular replacement (see Table S1).

Fluorescence Polarization DUB Assay

Ub-based fluorescence polarization substrates were used as previously described (Geurink et al., 2012).

Ub Chain Restriction Analysis

DUBs were diluted to 2× indicated concentrations, mixed with substrate, and incubated for 15 min at 37°C. Reactions were stopped by adding 4× lithium dodecyl sulfate sample buffer, resolved on 4%–12% SDS-PAGE gradient gels, and analyzed by silver staining and/or western blotting. Protocols for the generation of model substrates are described in detail in the Extended Experimental Procedures.

ACCESSION NUMBERS

Coordinates and structure factors have been deposited with the Protein Data Bank under the following accession numbers: OTUD1, 4BOP; OTUD2, 4BOQ; OTUD3, 4BOU; OTUD2-Lys11-diUb, 4BOZ; and OTUD2 Ub~K11 peptide, 4BOS.

SUPPLEMENTAL INFORMATION

Supplemental Information includes Extended Experimental Procedures, six figures, one data set, and one table and can be found with this article online at http://dx.doi.org/10.1016/j.cell.2013.05.046.

ACKNOWLEDGMENTS

We would like to thank E. Bennett (University of California, San Diego) for sharing unpublished mass spectrometry data, K. Bianchi and P. Meier (the Institute of Cancer Research, London) for the RIP1 purification protocol, T. Mund (Medical Research Council [MRC] Laboratory of Molecular Biology) for

providing GST-tagged NEDD4 and E6AP, and members of the D.K. lab for reagents and helpful discussions. This work was supported by the MRC (U105192732 to D.K.), the European Research Council (309756 to D.K.), the European Molecular Biology Organization Young Investigator program (to D.K.), the Lister Institute for Preventive Medicine (to D.K.), the Netherlands Organization for Scientific Research (700.58.011 to H.O.), and the Marie Curie Initial Training Network UPStream (to T.E.T.M.). Crystallographic data were collected at the European Synchrotron Radiation Facility at beam lines ID23-1 and ID29 and at the Diamond Light Source beam lines I-03 and I-04. H.O. and F.E. are cofounders of UbiQ Bio BV.

Received: December 9, 2012
Revised: May 2, 2013
Accepted: May 28, 2013
Published: July 3, 2013

REFERENCES

Akutsu, M., Ye, Y., Virdee, S., Chin, J.W., and Komander, D. (2011). Molecular basis for ubiquitin and ISG15 cross-reactivity in viral ovarian tumor domains. Proc. Natl. Acad. Sci. USA *108*, 2228–2233.

Borodovsky, A., Ovaa, H., Kolli, N., Gan-Erdene, T., Wilkinson, K.D., Ploegh, H.L., and Kessler, B.M. (2002). Chemistry-based functional proteomics reveals novel members of the deubiquitinating enzyme family. Chem. Biol. *9*, 1149–1159.

Bremm, A., Freund, S.M.V., and Komander, D. (2010). Lys11-linked ubiquitin chains adopt compact conformations and are preferentially hydrolyzed by the deubiquitinase Cezanne. Nat. Struct. Mol. Biol. *17*, 939–947.

Chen, Z.J., and Sun, L.J. (2009). Nonproteolytic functions of ubiquitin in cell signaling. Mol. Cell *33*, 275–286.

Clague, M.J., Coulson, J.M., and Urbé, S. (2012). Cellular functions of the DUBs. J. Cell Sci. *125*, 277–286.

Cooper, E.M., Cutcliffe, C., Kristiansen, T.Z., Pandey, A., Pickart, C.M., and Cohen, R.E. (2009). K63-specific deubiquitination by two JAMM/MPN+ complexes: BRISC-associated Brcc36 and proteasomal Poh1. EMBO J. *28*, 621–631.

Drag, M., Mikolajczyk, J., Békés, M., Reyes-Turcu, F.E., Ellman, J.A., Wilkinson, K.D., and Salvesen, G.S. (2008). Positional-scanning fluorigenic substrate libraries reveal unexpected specificity determinants of DUBs (deubiquitinating enzymes). Biochem. J. *415*, 367–375.

Edelmann, M.J., Iphöfer, A., Akutsu, M., Altun, M., di Gleria, K., Kramer, H.B., Fiebiger, E., Dhe-Paganon, S., and Kessler, B.M. (2009). Structural basis and specificity of human otubain 1-mediated deubiquitination. Biochem. J. *418*, 379–390.

Ekkebus, R., van Kasteren, S.I., Kulathu, Y., Scholten, A., Berlin, I., Geurink, P.P., de Jong, A., Goerdayal, S., Neefjes, J., Heck, A.J.R., et al. (2013). On terminal alkynes that can react with active-site cysteine nucleophiles in proteases. J. Am. Chem. Soc. *135*, 2867–2870.

Ernst, R., Mueller, B., Ploegh, H.L., and Schlieker, C. (2009). The otubain YOD1 is a deubiquitinating enzyme that associates with p97 to facilitate protein dislocation from the ER. Mol. Cell *36*, 28–38.

Faesen, A.C., Luna-Vargas, M.P.A., Geurink, P.P., Clerici, M., Merkx, R., van Dijk, W.J., Hameed, D.S., El Oualid, F., Ovaa, H., and Sixma, T.K. (2011). The differential modulation of USP activity by internal regulatory domains, interactors and eight ubiquitin chain types. Chem. Biol. *18*, 1550–1561.

Frias-Staheli, N., Giannakopoulos, N.V., Kikkert, M., Taylor, S.L., Bridgen, A., Paragas, J., Richt, J.A., Rowland, R.R., Schmaljohn, C.S., Lenschow, D.J., et al. (2007). Ovarian tumor domain-containing viral proteases evade ubiquitin- and ISG15-dependent innate immune responses. Cell Host Microbe *2*, 404–416.

Gerlach, B., Cordier, S.M., Schmukle, A.C., Emmerich, C.H., Rieser, E., Haas, T.L., Webb, A.I., Rickard, J.A., Anderton, H., Wong, W.W.-L., et al. (2011). Linear ubiquitination prevents inflammation and regulates immune signalling. Nature *471*, 591–596.

Geurink, P.P., El Oualid, F., Jonker, A., Hameed, D.S., and Ovaa, H. (2012). A general chemical ligation approach towards isopeptide-linked ubiquitin and ubiquitin-like assay reagents. ChemBioChem *13*, 293–297.

Hershko, A., and Ciechanover, A. (1998). The ubiquitin system. Annu. Rev. Biochem. *67*, 425–479.

Hospenthal, M.K., Freund, S.M.V., and Komander, D. (2013). Assembly, analysis and architecture of atypical ubiquitin chains. Nat. Struct. Mol. Biol. *20*, 555–565.

Hu, H., Brittain, G.C., Chang, J.-H., Puebla-Osorio, N., Jin, J., Zal, A., Xiao, Y., Cheng, X., Chang, M., Fu, Y.-X., et al. (2013). OTUD7B controls non-canonical NF-κB activation through deubiquitination of TRAF3. Nature *494*, 371–374.

Huang, O.W., Ma, X., Yin, J., Flinders, J., Maurer, T., Kayagaki, N., Phung, Q., Bosanac, I., Arnott, D., Dixit, V.M., et al. (2012). Phosphorylation-dependent activity of the deubiquitinase DUBA. Nat. Struct. Mol. Biol. *19*, 171–175.

Hymowitz, S.G., and Wertz, I.E. (2010). A20: from ubiquitin editing to tumour suppression. Nat. Rev. Cancer *10*, 332–341.

Iwai, K. (2011). Linear polyubiquitin chains: a new modifier involved in NFκB activation and chronic inflammation, including dermatitis. Cell Cycle *10*, 3095–3104.

Juang, Y.-C., Landry, M.-C., Sanches, M., Vittal, V., Leung, C.C.Y., Ceccarelli, D.F., Mateo, A.-R.F., Pruneda, J.N., Mao, D.Y.L., Szilard, R.K., et al. (2012). OTUB1 co-opts Lys48-linked ubiquitin recognition to suppress E2 enzyme function. Mol. Cell *45*, 384–397.

Kayagaki, N., Phung, Q., Chan, S., Chaudhari, R., Quan, C., O'Rourke, K.M., Eby, M., Pietras, E., Cheng, G., Bazan, J.F., et al. (2007). DUBA: a deubiquitinase that regulates type I interferon production. Science *318*, 1628–1632.

Keusekotten, K., Elliott, P.R., Glockner, L., Fiil, B.K., Damgaard, R.B., Kulathu, Y., Wauer, T., Gyrd-Hansen, M., Krappmann, D., Hofmann, K., et al. (2013). OTULIN Antagonizes LUBAC Signaling by Specifically Hydrolyzing Met1-Linked Polyubiquitin. Cell *153*, 1312–1326.

Kim, W., Bennett, E.J., Huttlin, E.L., Guo, A., Li, J., Possemato, A., Sowa, M.E., Rad, R., Rush, J., Comb, M.J., et al. (2011). Systematic and quantitative assessment of the ubiquitin-modified proteome. Mol. Cell *44*, 325–340.

Komander, D., and Rape, M. (2012). The ubiquitin code. Annu. Rev. Biochem. *81*, 203–229.

Komander, D., Clague, M.J., and Urbé, S. (2009). Breaking the chains: structure and function of the deubiquitinases. Nat. Rev. Mol. Cell Biol. *10*, 550–563.

Kulathu, Y., and Komander, D. (2012). Atypical ubiquitylation - the unexplored world of polyubiquitin beyond Lys48 and Lys63 linkages. Nat. Rev. Mol. Cell Biol. *13*, 508–523.

Licchesi, J.D.F., Mieszczanek, J., Mevissen, T.E.T., Rutherford, T.J., Akutsu, M., Virdee, S., El Oualid, F., Chin, J.W., Ovaa, H., Bienz, M., and Komander, D. (2012). An ankyrin-repeat ubiquitin-binding domain determines TRABID's specificity for atypical ubiquitin chains. Nat. Struct. Mol. Biol. *19*, 62–71.

Messick, T.E., Russell, N.S., Iwata, A.J., Sarachan, K.L., Shiekhattar, R., Shanks, J.R., Reyes-Turcu, F.E., Wilkinson, K.D., and Marmorstein, R. (2008). Structural basis for ubiquitin recognition by the Otu1 ovarian tumor domain protein. J. Biol. Chem. *283*, 11038–11049.

Nakada, S., Tai, I., Panier, S., Al-Hakim, A., Iemura, S.-I., Juang, Y.-C., O'Donnell, L., Kumakubo, A., Munro, M., Sicheri, F., et al. (2010). Non-canonical inhibition of DNA damage-dependent ubiquitination by OTUB1. Nature *466*, 941–946.

Nakasone, M.A., Livnat-Levanon, N., Glickman, M.H., Cohen, R.E., and Fushman, D. (2013). Mixed-linkage ubiquitin chains send mixed messages. Structure *21*, 727–740.

Reyes-Turcu, F.E., Ventii, K.H., and Wilkinson, K.D. (2009). Regulation and cellular roles of ubiquitin-specific deubiquitinating enzymes. Annu. Rev. Biochem. *78*, 363–397.

Rumpf, S., and Jentsch, S. (2006). Functional division of substrate processing cofactors of the ubiquitin-selective Cdc48 chaperone. Mol. Cell *21*, 261–269.

Schaefer, J.B., and Morgan, D.O. (2011). Protein-linked ubiquitin chain structure restricts activity of deubiquitinating enzymes. J. Biol. Chem. *286*, 45186–45196.

Sowa, M.E., Bennett, E.J., Gygi, S.P., and Harper, J.W. (2009). Defining the human deubiquitinating enzyme interaction landscape. Cell *138*, 389–403.

Tsai, Y.C., and Weissman, A.M. (2011). Ubiquitylation in ERAD: reversing to go forward? PLoS Biol. *9*, e1001038.

Wagner, S.A., Beli, P., Weinert, B.T., Nielsen, M.L., Cox, J., Mann, M., and Choudhary, C. (2011). A proteome-wide, quantitative survey of in vivo ubiquitylation sites reveals widespread regulatory roles. Mol Cell Proteomics *10*, M111.013284.

Wang, Y., Satoh, A., Warren, G., and Meyer, H.H. (2004). VCIP135 acts as a deubiquitinating enzyme during p97-p47-mediated reassembly of mitotic Golgi fragments. J. Cell Biol. *164*, 973–978.

Wang, T., Yin, L., Cooper, E.M., Lai, M.-Y., Dickey, S., Pickart, C.M., Fushman, D., Wilkinson, K.D., Cohen, R.E., and Wolberger, C. (2009). Evidence for bidentate substrate binding as the basis for the K48 linkage specificity of otubain 1. J. Mol. Biol. *386*, 1011–1023.

Wickliffe, K.E., Williamson, A., Meyer, H.-J., Kelly, A., and Rape, M. (2011). K11-linked ubiquitin chains as novel regulators of cell division. Trends Cell Biol. *21*, 656–663.

Wiener, R., Zhang, X., Wang, T., and Wolberger, C. (2012). The mechanism of OTUB1-mediated inhibition of ubiquitination. Nature *483*, 618–622.

Williamson, A., Banerjee, S., Zhu, X., Philipp, I., Iavarone, A.T., and Rape, M. (2011). Regulation of ubiquitin chain initiation to control the timing of substrate degradation. Mol. Cell *42*, 744–757.

Williamson, A., Werner, A., and Rape, M. (2013). The Colossus of ubiquitylation: decrypting a cellular code. Mol. Cell *49*, 591–600.

Ye, Y., Akutsu, M., Reyes-Turcu, F., Enchev, R.I., Wilkinson, K.D., and Komander, D. (2011). Polyubiquitin binding and cross-reactivity in the USP domain deubiquitinase USP21. EMBO Rep. *12*, 350–357.

Ye, Y., Blaser, G., Horrocks, M.H., Ruedas-Rama, M.J., Ibrahim, S., Zhukov, A.A., Orte, A., Klenerman, D., Jackson, S.E., and Komander, D. (2012). Ubiquitin chain conformation regulates recognition and activity of interacting proteins. Nature *492*, 266–270.

EXTENDED EXPERIMENTAL PROCEDURES

Cloning and Molecular Biology

Full-length OTU DUBs were cloned from IMAGE clones, cDNA libraries, or from plasmids that were kinds gifts from C. Schlieker (Yale University, OTUD2), B. Kessler (University of Oxford, OTUB1), M. Balakirev (CEA Grenoble, OTUB2), R. Marmorstein (Wistar Institute, yOtu1), S. Todi (Wayne State University, dmOtu1), S. Urbe (University of Liverpool, VCPIP). DNA sequences to generate proteins listed in Figure 1C were PCR amplified using KOD HotStart polymerase (Novagen) and cloned into pOPIN (Berrow et al., 2007) vectors using the In-Fusion HD system (Clontech) according to the manufacturer protocol. Mutagenesis was performed using the QuikChange protocol, but using KOD polymerase according to manufacturer's protocol.

Protein Expression and Purification from Bacteria

For bacterial production, protein was expressed from pOPIN-K vectors, which introduces a PreScission protease cleavable N-terminal His6-GST tag. Protein was expressed in *E. coli* Rosetta2 pLacI cells that were grown to an OD_{600} of 0.8-1.0 at 37°C and induced with 1 mM IPTG for 16-20 hr at 20°C. Large-scale protein expression was performed in 2-12 L LB or 2xTY medium supplemented with the appropriate antibiotics.

Protein purifications were performed at 4°C. Cells were lysed by sonication in 50-100 ml lysis buffer (200 mM NaCl, 25 mM Tris [pH8.5], 5 mM DTT, 1 EDTA free Complete protease inhibitor tablet, 0.1 mg/mL DNase, 1 mg/mL lysozyme), and cleared by centrifugation 35000 rpm for 45 min. The cleared lysate was incubated with 1.5-3 ml equilibrated Glutathione Sepharose 4B resin (GE Healthcare) for 1 hr, and subsequently washed with 2 L buffer A (25 mM Tris [pH 8.5], 5 mM DTT) plus 500 mM NaCl, and 500 ml buffer A plus 50 mM NaCl. The GST tag was cleaved on the resin with 50 μg GST-tagged PreScission protease overnight. Cleaved protein was eluted with buffer A plus 50 mM NaCl to a final volume of 50 mL, and subjected to anion exchange chromatography (ResourceQ 1mL, GE Healthcare). OTU DUBs usually elute as a single peak in buffer A with a NaCl gradient from 50 to 500 mM. Peak fractions were pooled and subjected to gel filtration (Superdex75) in buffer A plus 200 mM NaCl. Proteins were concentrated to 2-20 mg/mL using a VivaSpin 10 kDa MW cut-off concentrator and flash-frozen in liquid nitrogen.

OTUD5 (aa 171-358) was catalytically inactive when produced in *E.coli*, as reported (Huang et al., 2012). Incubation of 500 μg OTUD5 (10 mg/mL) with 150 U of casein kinase 2 (CK2) in 20 mM Tris (pH 7.5), 50 mM KCl, 10 mM $MgCl_2$, 10 mM ATP for 4 hr at 30°C resulted in phosphorylation and activation of the enzyme. The reaction was stopped by addition of 20 mM EDTA (final concentration) and used for DUB assays.

Modification of OTU DUBs by Suicide Probes

Generation of Ub-, NEDD8- and ISG15-derived chemical probes was performed according to published protocols (Akutsu et al., 2011; Borodovsky et al., 2002). Purified Ub- and Ubl-thioesters were diluted with DUB dilution buffer (150 mM NaCl, 25 mM Tris (pH 7.5), and 10 mM DTT) to a final concentration of 2 mg/mL. 200 μl Ub-/Ubl-thioester was mixed with 40 mg 2-bromoethylamine hydrochloride solved in 200 μl phosphate buffered saline (PBS [pH 4.8]). The reaction was initiated by adding 80 μl 2 M NaOH and incubated for 15 min on ice. Subsequent dialysis against probe buffer (200 mM NaCl, 25 mM Tris [pH 7.5]) was performed using Slide-A-Lyzer Dialysis Cassettes (Thermo Scientific).

Ub propargylamide (Ub-PA) and Cy5-labeled Ub-PA were generated according to (Ekkebus et al., 2013). Ub-PA probes were dissolved in DMSO, diluted to 1.7 mg/mL in H_2O and 1:1 mixed with 2x probe buffer.

For suicide probe assays, 8 μl of OTU DUB diluted to 0.4 mg/mL in DUB dilution buffer, was mixed with 20 μl Ub-, NEDD8- or ISG15-derived probes at 0.83 mg/mL. After incubation at room temperature for indicated times, the reaction was stopped by addition of 10 μl 4x LDS sample buffer (Invitrogen). 13 μl of each sample was resolved by SDS-PAGE prior to staining using Instant Blue SafeStain (Expedeon). vOTU (Akutsu et al., 2011) was used as a positive control as it is readily modified with each probe (Figures 1 and S1).

In Vitro DUB Assays

Qualitative in vitro linkage specificity assays were performed as described in (Licchesi et al., 2012). In brief, a 2x reaction stock is prepared containing 2 μg diUb, 100 mM NaCl, 100 mM Tris (pH 7.5), 10 mM DTT. To this is added 10 μl of 2x concentrated DUB in DUB dilution buffer. DiUb concentrations are re-adjusted frequently. Pilot experiments identified the required DUB concentration that resulted in robust cleavage of the preferred chain type.

The assay is started by mixing DUB and diUb solutions, and 5 μl aliquots are taken at indicated time points. The reaction is resolved on 4%–12% SDS-PAGE gradient gels run in MES buffer (Invitrogen) and stained by silver staining using the SilverStain Plus kit (BioRad).

NMR Analysis of the OTUD2 ZnF Domain

Expression of ^{13}C, ^{15}N-labeled OTUD2 ZnF constructs was performed in 2 L M9 minus medium supplemented with 2 mM $MgSO_4$, 50 μM $ZnCl_2$, 10 μM $CaCl_2$ (all final concentrations), 10 ml *E. coli* trace elements, 4 g ^{13}C-glucose, 2 g ^{15}N-labeled NH_4Cl and antibiotics. Cultures were grown at 37°C to an $O.D._{600}$ of 0.8-1.0, and cooled down to 20°C prior to induction with 1 mM IPTG. 200 μM $ZnSO_4$ was added at the time of induction. GST-tagged protein was purified as described above, and stored in NMR buffer (18 mM

Na_2HPO_4, 7 mM NaH_2PO_4 x H_2O, 150 mM NaCl, 5 mM DTT [pH 7.2]). Unlabeled proteins used for NMR studies were dialyzed against NMR buffer. Samples were prepared in 500 μl NMR buffer, supplemented with 35 μl D_2O and transferred to a NMR tube.

NMR acquisition was carried out at 298 K on a Bruker Avance 600 MHz spectrometer equipped with a TCI triple resonance cryoprobe. In order to assign the ZnF domain of OTUD2, standard triple resonance experiments (CBCA(CO)NH and HNCACB) were acquired. For chemical shift perturbation experiments, HSQC (Heteronuclear Single Quantum Coherence) spectra were recorded for 50 μM ^{13}C, ^{15}N-labeled OTUD2 ZnF alone and in the presence of 1 mM unlabeled Ub. The reverse experiment was performed with 80 μM ^{13}C, ^{15}N-labeled Ub alone and with 400 μM unlabeled OTUD2 ZnF.

Data processing and analysis were carried out in TopSpin (Bruker BioSpin) and Sparky (Goddard TD & Kneller DG, University of California, San Francisco), respectively. The full backbone of OTUD2 ZnF was assigned using MARS (Jung and Zweckstetter, 2004).

Crystallization of OTU DUBs

Crystallization screening was performed using nano-liter robotics (typical volume 100+100 nL) in a sitting drop setup, using up to 1800 conditions from commercial screens per protein. Most crystal hits were reproduced in hanging-drop setup. OTUD1 crystals grew at 20°C from Morpheus screen (Gorrec, 2009) condition C7, containing 10% (v/v) PEG 4000, 0.1 M Buffer 2, 0.09 M NPS, 20% (v/v) glycerol. The small crystals were vitrified in liquid nitrogen. OTUD2 crystals grew at 20°C from 0.8 M sodium phosphate monobasic monohydrate, 0.1 M Tris [pH 7.5]. For synchrotron data collection, crystals were soaked in mother liquor containing 30% glycerol, and vitrified in liquid nitrogen. OTUD3 crystals grew from 10% (v/v) PEG 400, 0.1 M KCl, 0.01 M MgCl, 50 mM MES (pH 6.0). Crystals were cryo-cooled after brief soaking in mother liquor containing 15% (v/v) glycerol. OTUD2 C160 A in complex with Lys11-linked diUb grew from 0.15 M potassium thiocyanate, 18% (w/v) PEG 5000 MME, 0.1 M sodium acetate (pH 5.5). Crystals were soaked in mother liquor containing 30% glycerol prior to data collection. OTUD2 C160A in complex with a ubiquitinated peptide were grown in 21% (v/v) PEG 3350, 100 mM NaOAc (pH 5.6), 200 mM $Mg(NO_3)_2$. Crystals were transferred into paratone-N-oil prior to vitrification in liquid nitrogen.

Data Collection, Phasing, and Refinement

Diffraction data were collected at the ESRF (Grenoble, FR), beam lines ID23-1 and ID29, and at the Diamond Light Source, beam lines I-03 and I-04. OTUD1 crystals were in space group *I*4 with two molecules in the asymmetric unit (mol/AU) and diffracted to 2.1 Å resolution. OTUD2 apo crystals were in space group $P3_121$ with one mol/AU and diffracted to 1.5 Å resolution. OTUD3 crystals were in space group $P3_212$ and diffracted to 1.55 Å resolution with 1 mol/AU. OTUD2 C160A crystals in complex with Lys11-linked diUb were in space group C2, diffracted to 3.0 Å and contained 2 OTUD2 and 3 Ub molecules in the AU. OTUD2 crystals in complex with ubiquitinated K11 peptide were in space group *P*6 with 2 OTUD2 and 2 Ub molecules in the AU and diffracted to 2.35 Å (see Table S1).

Phases were obtained by molecular replacement in Phaser (McCoy et al., 2007), using refined structures as listed in Table S1 as search models. Balbes (Long et al., 2008) identified a fragment of OTUD5 lacking the N-terminal helix as the best search model for OTUD3, and this was used in molecular replacement in Phaser. For high resolution structures (OTUD1, OTUD2, OTUD3), initial models were automatically built by WarpNTrace (Langer et al., 2008). Structures were built in Coot (Emsley and Cowtan, 2004) and refined in PHENIX (Adams et al., 2002) or Refmac (Murshudov et al., 2011), including simulated annealing and TLS B-factor refinement where appropriate. In the case of OTUD3, B-factors were refined with anisotropic restraints. Final statistics for all structures can be found in Table S1.

Fluorescence Polarization DUB Assay

The Ub-based FP substrates and peptides were used as described in (Faesen et al., 2011) and (Geurink et al., 2012). FP assays were performed using a Pherastar plate reader (BMG Labtech) equipped with a 550 nm excitation filter and two 590 nm emission filters. Fluorescence intensities were measured in the S (parallel) and P (perpendicular) direction. FP values are given in mP (millipolarization) and calculated using the Equation 1:

$$Polarization(mP) = \frac{S-(G\cdot P)}{S+(G\cdot P)} \times 1000 \qquad \text{(Equation 1)}$$

The confocal optics were adjusted with the average P and S values for TAMRA-KG and the grating factor (G) was determined using a polarization value (L) for TAMRA-KG (25 nM) of 50 mP using Equation 2:

$$G = \frac{average\ S}{average\ P} \times \frac{1-\left(\frac{L}{1000}\right)}{1+\left(\frac{L}{1000}\right)} \qquad \text{(Equation 2)}$$

The assays were performed in "non binding surface flat bottom low flange" black 384-well plates (Corning) at room temperature in a buffer containing 20 mM TrisHCl, pH 7.5, 5 mM DTT, 100 mM NaCl, 1 mg/mL 3-[(3-cholamidopropyl) dimethylammonio] propanesulfonic acid (CHAPS) and 0.5 mg/mL bovine gamma globulin (BGG). Each well had a volume of 20 μL. Buffer and enzyme (OTUD1, OTUD2, OTUD3, OTUB1, Cezanne2) were predispensed (10 μL/well) and the reaction was started by the addition of substrate

(10 µL/well, 5 µM final concentration (0.5 µM for OTUD1)). The plate was centrifuged (1 min at 1,500 rpm) prior to the measurement. Kinetic data were collected in intervals of 30 s. The obtained data were fitted according to a ‘one phase exponential decay’ using Prism 5.01 (GraphPad Software).

Generation of Model Substrates for Ub Chain Restriction Analysis

Lys63-polyUb was assembled using TRAF6/UBE2N/UBE2V1 (Deng et al., 2000) and Lys11-polyUb using UBE2S according to (Bremm et al., 2010). GST-tagged E6AP and NEDD4 (kind gift from Thomas Mund, MRC LMB) were autoubiquitinated using 100 nM wheat E1, 2.3 µM UBE2L3, 10 µM E3, 50 µM Ub (Kim and Huibregtse, 2009). Met1-linked polyUb was assembled using 5 µM HOIP 699-1072, 100 nM wheat E1, 6 µM UBE2L3, 0.25 mg/mL Ub (Smit et al., 2012). All substrates except HOIP were purified on a AKTA Micro system to remove short free chains and monoUb.

TNF Receptor Purification

TNFRSC was purified from the indicated cell lines after stimulation with 100 ng/mL Flag-TNFα (Human TNFα, from Alexis) for 10 min. Ice-cold PBS was added to the plate to stop stimulation. Following lysis, TNFRSC was purified by incubation with Flag M2 agarose beads (Sigma). TNFR was purified from the unstimulated sample by adding 1 µg of TNFα during lysis. The purified TNFRSC was analyzed by western blotting for RIP1. Ub chain restriction analysis was performed on beads.

Ub Chain Restriction Analysis

DUBs were diluted to 2x indicated concentrations in 150 mM NaCl, 25 mM Tris (pH 7.5), and 10 mM DTT and activated at 23°C for 10 min. Subsequently, 5 µl of diluted enzyme were mixed with 5 µl substrate, incubated for 15 min at 37°C, and the reaction stopped by adding 10 µl 4x LDS sample buffer (Invitrogen). 1-10 µl of the reaction was resolved on 4%–12% SDS-PAGE gradient gels run in MES buffer, and visualized by silver staining (SilverStain Plus Kit, BioRad) or by western analysis using polyclonal anti-Ub antibody (Millipore).

SUPPLEMENTAL REFERENCES

Adams, P.D., Grosse-Kunstleve, R.W., Hung, L.W., Ioerger, T.R., McCoy, A.J., Moriarty, N.W., Read, R.J., Sacchettini, J.C., Sauter, N.K., and Terwilliger, T.C. (2002). PHENIX: building new software for automated crystallographic structure determination. Acta Crystallogr. D Biol. Crystallogr. *58*, 1948–1954.

Berrow, N.S., Alderton, D., Sainsbury, S., Nettleship, J., Assenberg, R., Rahman, N., Stuart, D.I., and Owens, R.J. (2007). A versatile ligation-independent cloning method suitable for high-throughput expression screening applications. Nucleic Acids Res. *35*, e45.

Deng, L., Wang, C., Spencer, E., Yang, L., Braun, A., You, J., Slaughter, C., Pickart, C., and Chen, Z.J. (2000). Activation of the IkappaB kinase complex by TRAF6 requires a dimeric ubiquitin-conjugating enzyme complex and a unique polyubiquitin chain. Cell *103*, 351–361.

Emsley, P., and Cowtan, K. (2004). Coot: model-building tools for molecular graphics. Acta Crystallogr. D Biol. Crystallogr. *60*, 2126–2132.

Gorrec, F. (2009). The MORPHEUS protein crystallization screen. J. Appl. Cryst. *42*, 1035–1042.

Jung, Y.-S., and Zweckstetter, M. (2004). Mars — robust automatic backbone assignment of proteins. J. Biomol. NMR *30*, 11–23.

Kim, H.C., and Huibregtse, J.M. (2009). Polyubiquitination by HECT E3s and the determinants of chain type specificity. Mol. Cell. Biol. *29*, 3307–3318.

Langer, G., Cohen, S.X., Lamzin, V.S., and Perrakis, A. (2008). Automated macromolecular model building for X-ray crystallography using ARP/wARP version 7. Nat. Protoc. *3*, 1171–1179.

Long, F., Vagin, A.A., Young, P., and Murshudov, G.N. (2008). BALBES: a molecular-replacement pipeline. Acta Crystallogr. D Biol. Crystallogr. *64*, 125–132.

McCoy, A.J., Grosse-Kunstleve, R.W., Adams, P.D., Winn, M.D., Storoni, L.C., and Read, R.J. (2007). Phaser crystallographic software. J. Appl. Cryst. *40*, 658–674.

Murshudov, G.N., Skubák, P., Lebedev, A.A., Pannu, N.S., Steiner, R.A., Nicholls, R.A., Winn, M.D., Long, F., and Vagin, A.A. (2011). REFMAC5 for the refinement of macromolecular crystal structures. Acta Crystallogr. D Biol. Crystallogr. *67*, 355–367.

Smit, J.J., Monteferrario, D., Noordermeer, S.M., van Dijk, W.J., van der Reijden, B.A., and Sixma, T.K. (2012). The E3 ligase HOIP specifies linear ubiquitin chain assembly through its RING-IBR-RING domain and the unique LDD extension. EMBO J. *31*, 3833–3844.

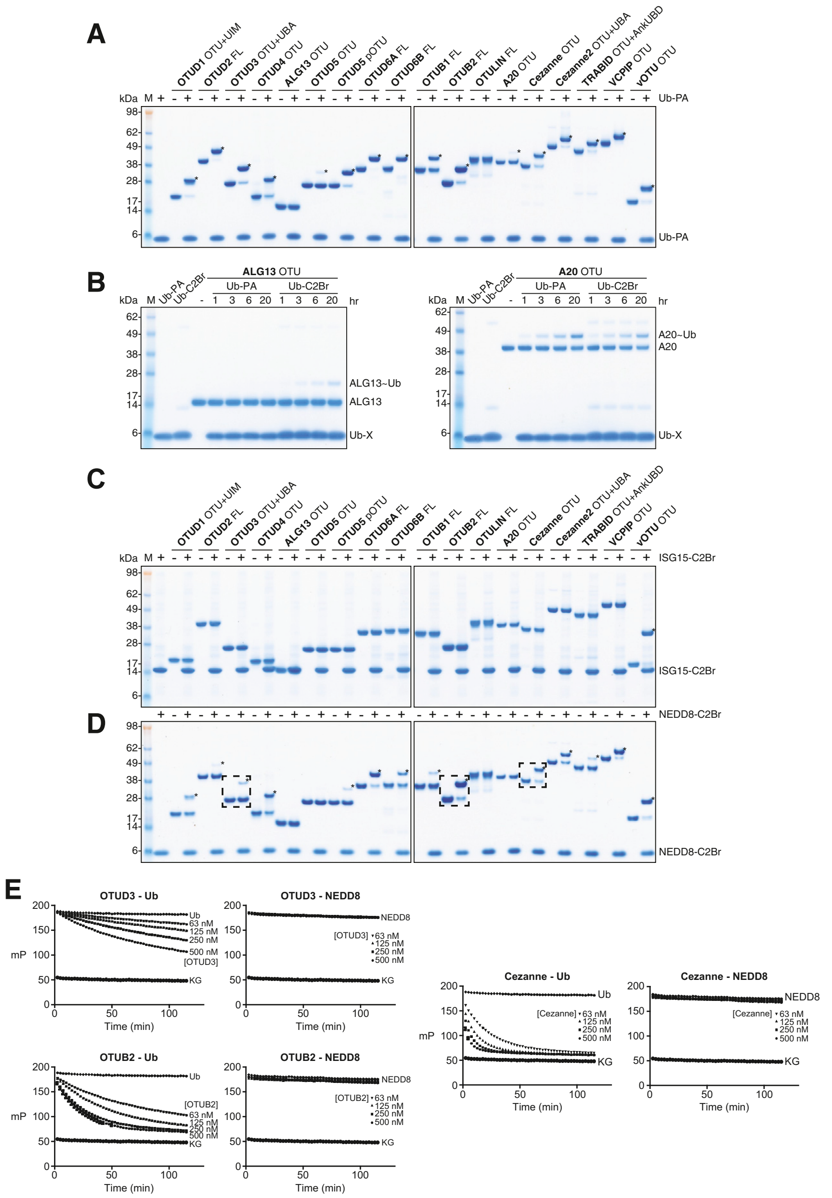

Figure S1. Reactivity of Human OTU DUBs with Probes Derived from Ub and Ub-like Modifiers, Related to Figure 1

(A) OTU DUBs shown alone and after incubation with the suicide probe Ub-propargylamide (Ub-PA, 1 hr reactions). Asterisks (*) indicate modified forms of DUB.

(B) Reactivity of ALG13 and A20, which did not react with Ub-PA (Figures 1E and S1A 1 hr reactions) against Ub-PA and Ub bromoethylamine (C2Br).

(C) Reactivity of OTU DUBs against ISG15-C2Br (3 hr reactions). Only vOTU is modified, indicated by an asterisk (*).

(D) Reactivity of OTU DUBs against NEDD8-C2Br (3 hr reactions). Asterisks (*) indicate NEDD8 modified enzymes.

(E) Fluorescence anisotropy assays of selected OTU domains (boxed in D) against fluorescent ubiquitinated or neddylated KG peptides. The raw data are shown at different enzyme concentrations.

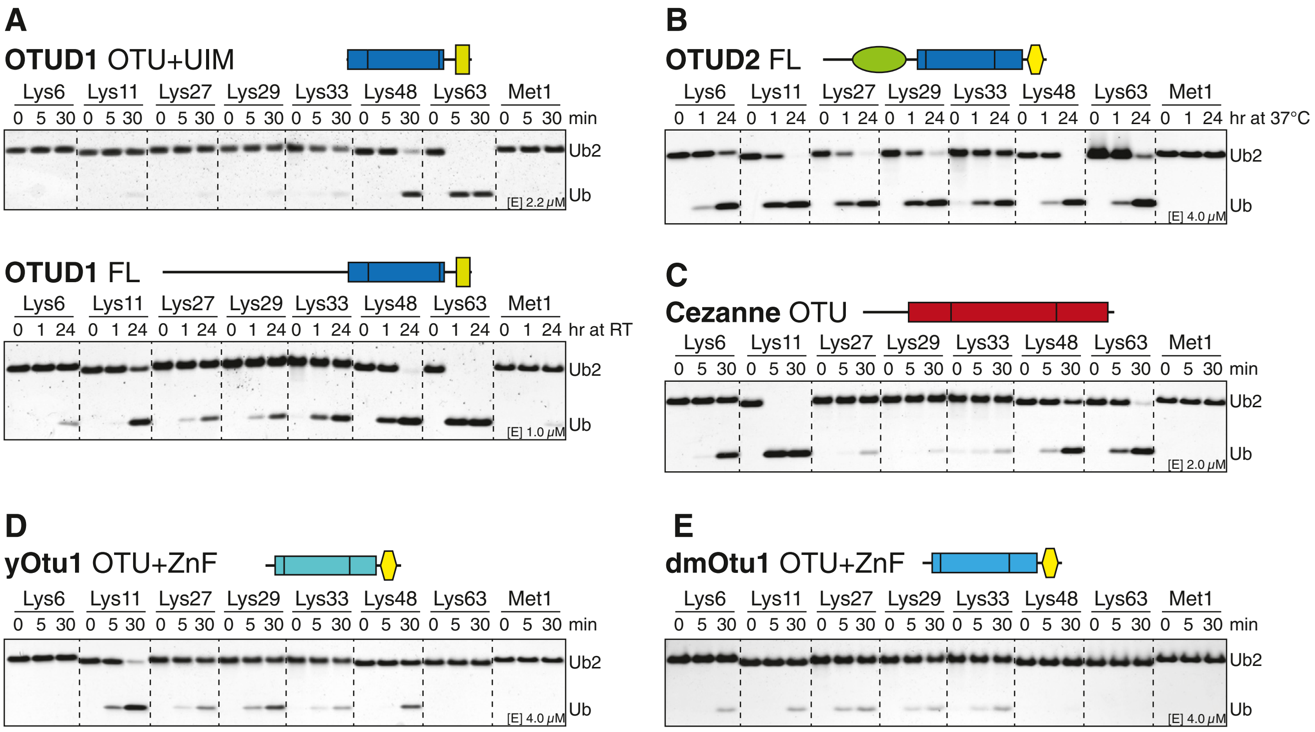

Figure S2. Additional DUB Assays for Selected OTU DUBs, Related to Figure 2

(A) OTUD1 constructs tested at 22× concentration (top) or at 10× concentration and longer time course (24 hr, *bottom*) compared to Figure 2A showing that under these conditions, other linkages are hydrolyzed.

(B) OTUD2 DUB assay at long time points (24 hr).

(C) DUB assay of Cezanne at 10× higher concentration reveals that all isopeptide-linked diUb are hydrolyzed.

(D) Linkage specificity of *S. cerevisiae* Otu1 (yOtu1), aa 91–301 (Messick et al., 2008). yOTU1 has a similar linkage specificity profile as its human counterpart OTUD2.

(E) DUB assay of *D. melanogaster* Otu1 (dmOtu1), aa 143–347, shows a similar cleavage pattern compared to human and yeast orthologs but additionally cleaves K6-linked diUb.

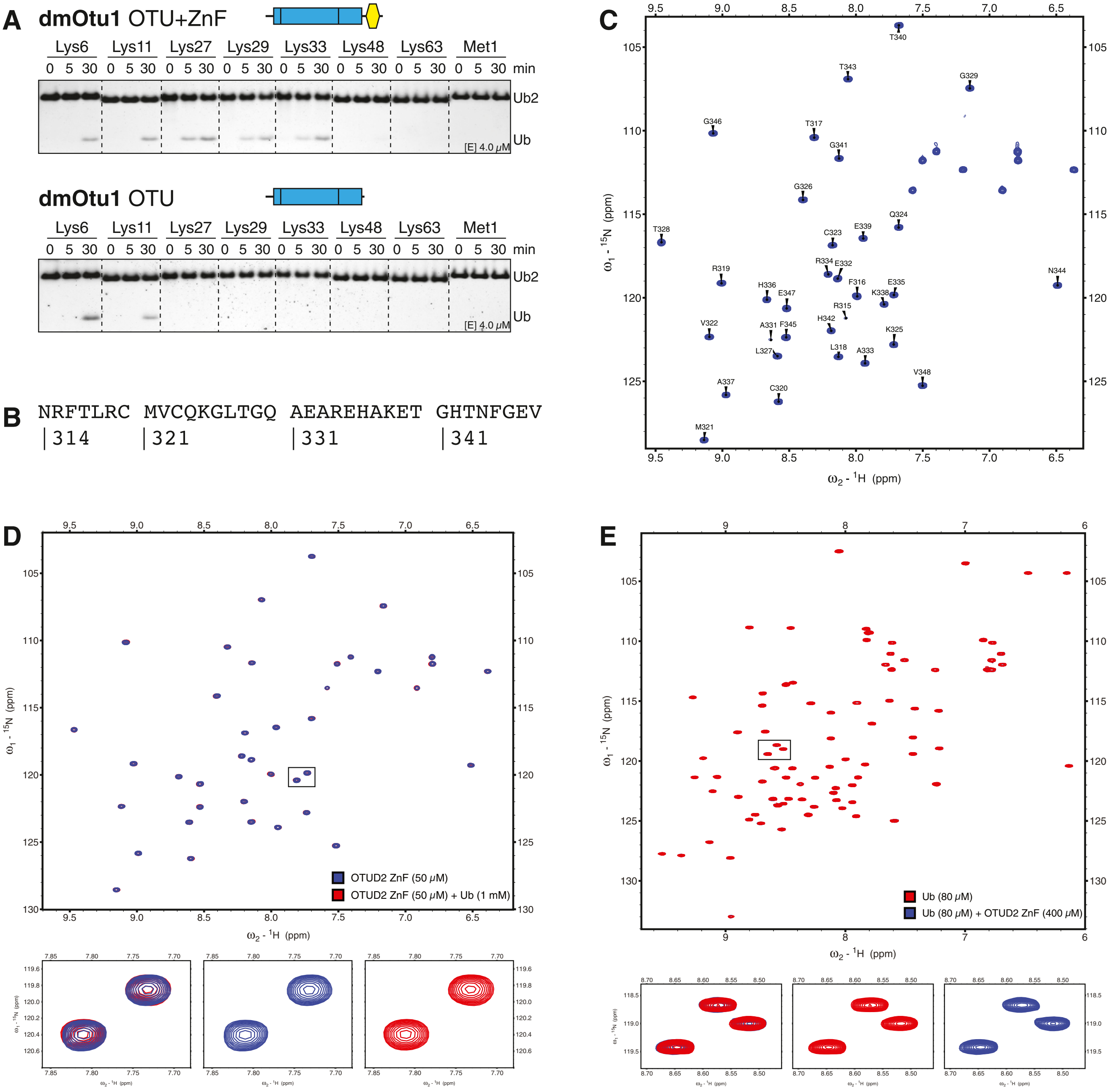

Figure S3. Impact of the ZnF Domain on dmOtu1 Specificity and the Ability of the Human OTUD2 ZnF Domain to Bind monoUb, Related to Figure 3

(A) Specificity analysis of different dmOtu1 constructs. *Top*, dmOtu1 lacking the UBX-like domain. *Bottom*, isolated catalytic OTU domain. As in the human ortholog OTUD2, the ZnF domain affects the enzyme's ability to cleave Lys27-, Lys29- and Lys33-linked diUb.

(B) Sequence of the analyzed human OTUD2 ZnF construct (aa 314–348).

(C) Fully assigned ZnF spectrum derived from 3D NMR experiments.

(D) Ub binding experiment with OTUD2 ZnF construct. Shown are overlaid spectra of 50 μM OTUD2 ZnF alone (blue) and with addition of 1 mM unlabeled Ub (red). The boxed section is shown in close-up view below. The lack of chemical shift perturbations indicate no binding of the ZnF to monoUb.

(E) The reverse experiment from (C), using 80 μM labeled Ub and 400 μM unlabeled OTUD2 ZnF. Again, no Ub residue is perturbed, hence there is no binding under these conditions.

Cell

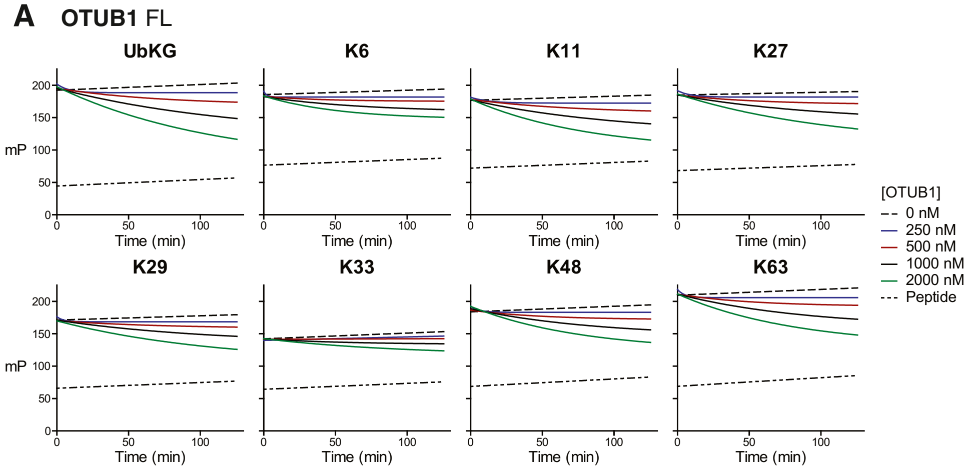

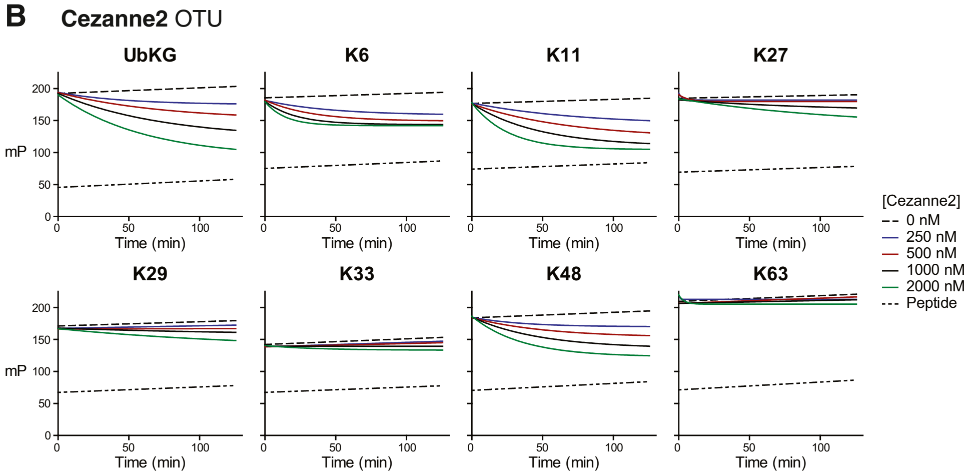

C **Ala scan**

Mutation	Peptide sequence
native	TAMRA-FVKTLTGK(Ub)TITLEV
F4A	TAMRA-AVKTLTGK(Ub)TITLEV
V5A	TAMRA-FAKTLTGK(Ub)TITLEV
T7A	TAMRA-FVKALTGK(Ub)TITLEV
L8A	TAMRA-FVKTATGK(Ub)TITLEV
T9A	TAMRA-FVKTLAGK(Ub)TITLEV
T12A	TAMRA-FVKTLTGK(Ub)AITLEV
I13A	TAMRA-FVKTLTGK(Ub)TATLEV
T14A	TAMRA-FVKTLTGK(Ub)TIALEV
L15A	TAMRA-FVKTLTGK(Ub)TITAEV
E16A	TAMRA-FVKTLTGK(Ub)TITLAV
V17A	TAMRA-FVKTLTGK(Ub)TITLEA

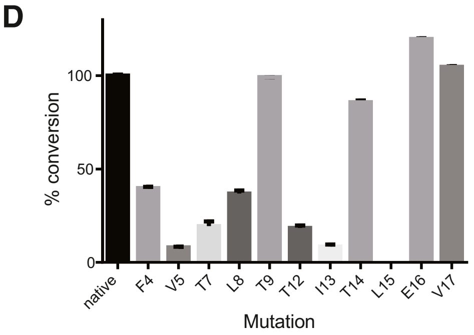

Figure S4. Additional Fluorescence Polarization Assays and Ala Scan of the K11 Peptide, Related to Figure 4

(A and B) Fluorescence polarization assays as in Figure 4B–4D for OTUB1 (A) and Cezanne2 (B).

(C and D) Alanine scanning mutagenesis of the K11 peptide.

(C) Sequences of mutated fluorescent DUB substrates.

(D) Bar graph representation of graphs shown in Figure 4F. Error bars represent SD from the mean.

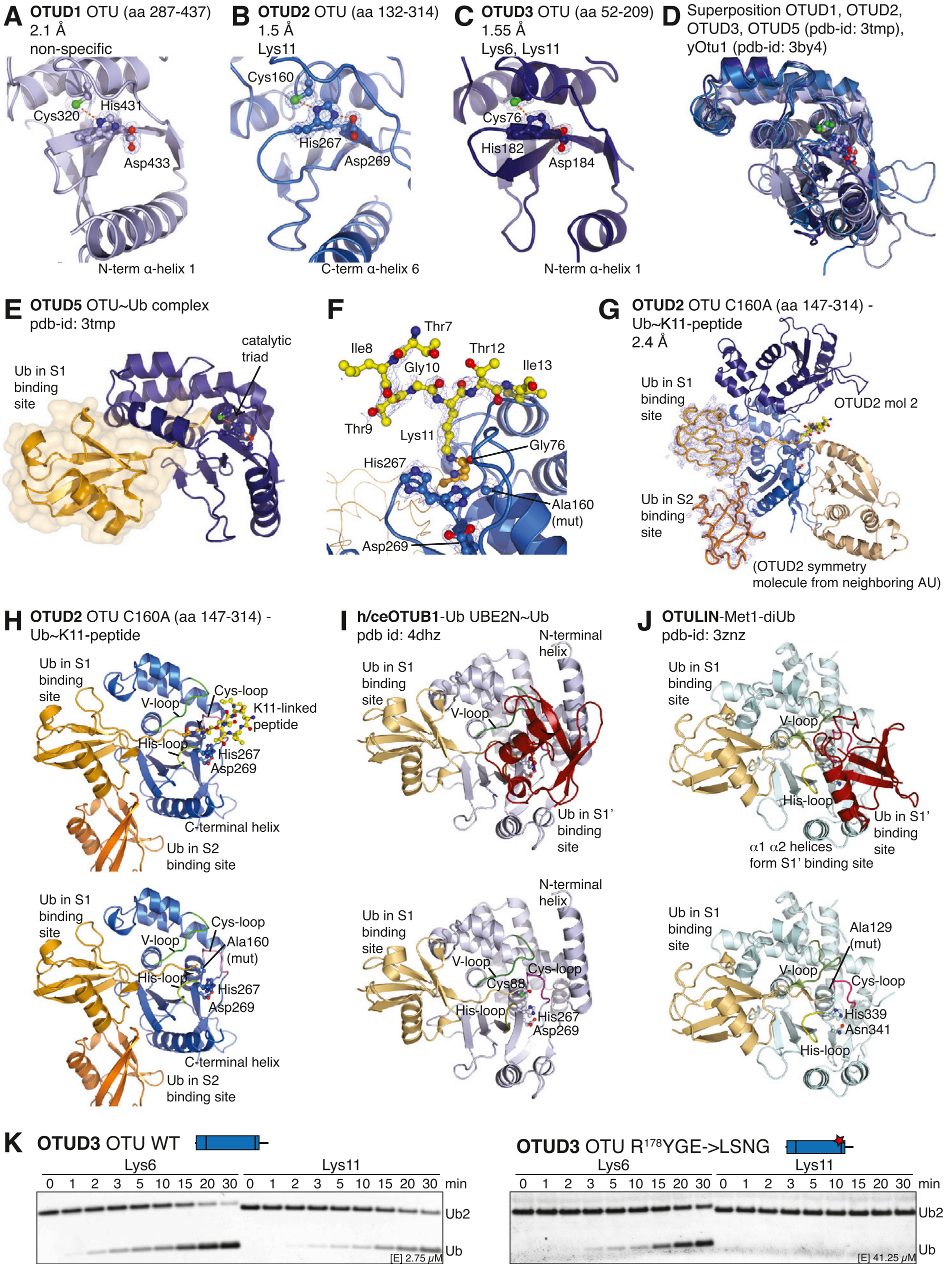

Figure S5. Structural Studies on OTUD1, OTUD2, and OTUD3 Reveals a Conserved S1' Site, Related to Figure 5

(A–C) Close-up view of OTUD1 (A), OTUD2 (B) and OTUD3 (C) with 2|Fo|-|Fc| electron density contoured at 1σ covering catalytic triad residues.

(D) Superposition of OTUD1, OTUD2, OTUD3, OTUD5/DUBA (Huang et al., 2012) and yOtu1 (Messick et al., 2008). Structures are highly similar with low rmsd values (~0.8 Å).

(E) Structure of phosphorylated human OTUD5 bound to a Ub suicide probe (pdb-id 3tmp) (Huang et al., 2012).

(F) 2|Fo|-|Fc| electron density contoured at 1σ covers the isopeptide and peptide bound across the active site of OTUD2.

(G) Arrangement of molecules in the OTUD2 C160A complex with ubiquitinated K11 peptide. Two OTUD2 molecules are present, one of which interacts with two Ub moieties in S1 and S2 sites. 2|Fo|-|Fc| electron density contoured at 1σ covers the Ub molecules in the complex, indicating that the molecule in the S2 site is less well ordered. A beige OTUD2 from a neighboring asymmetric unit forms a crystal contact with the complexed OTUD2 molecule, affecting the peptide binding site of OTUD2.

(H–J) OTUD2 C160A complex with ubiquitinated K11 peptide (H), the OTUB1 structure with two Ub moieties bound in S1 and S1' sites (Wiener et al., 2012) (I) and OTULIN in complex with Met1-linked diUb (J) in identical orientations. Putative S1' site elements (Cys, His, and V loop C-terminal helix in OTUD2) are indicated.

(K) A His-lop mutation in OTUD3, R^{178}YGE to LSNG creates a OTU DUB with reduced activity against Lys11-linked diUb. Comparison of a fine time course of Lys6- and Lys11-linked diUb. Note differences in enzyme concentration used in assays.

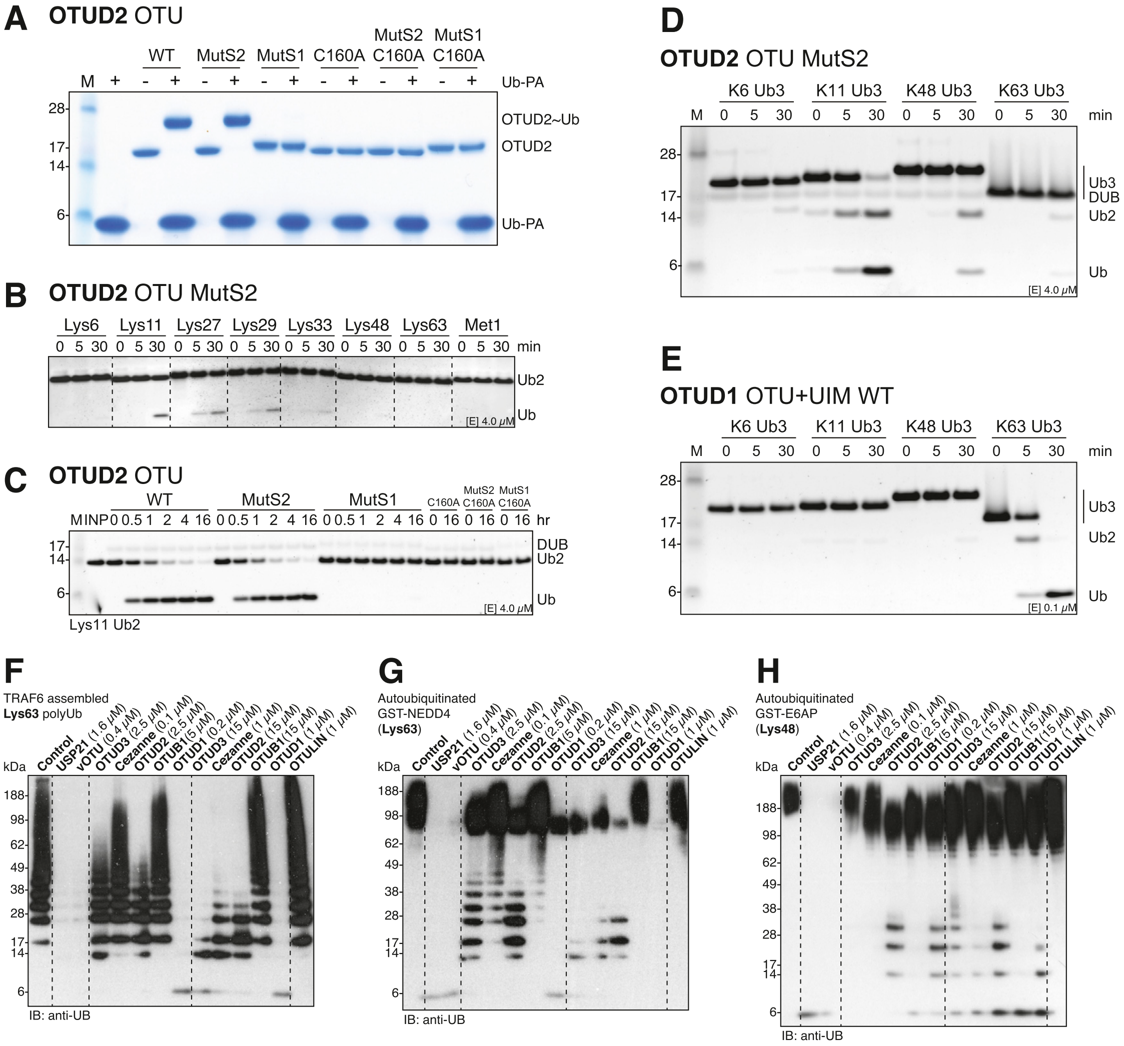

Figure S6. Additional Assays for S2 Site Characterization and Ub Chain Restriction Analysis, Related to Figures 6 and 7

(A) Reactivity of wild-type OTUD2 catalytic domain (aa 147–314), S2 site mutant (MutS2, aa 147–314, I292Q, V295Q), S1 site mutant (MutS1, aa 147–314, AI200-201DD) and corresponding inactive variants (C160A) against Ub-PA.

(B) Specificity analysis of OTUD2 MutS2.

(C) DUB assay with constructs in (A) against K11-linked diUb.

(D) Cleavage of triUb substrates as in (B).

(E) Specificity analysis of OTUD1 using triUb substrates.

(F–H) Western blotted restriction analysis gels (anti-Ub) of GST-TRAF6 assembled free and attached Lys63-linked polyUb (F), autoubiquitinated GST-NEDD4 (G) and GST-E6AP (H). Compare silver-stained counterparts in Figures 7C–7E.
